# Reproductive assurance drives transitions to self-fertilization in experimental *Caenorhabditis elegans*

**DOI:** 10.1186/s12915-014-0093-1

**Published:** 2014-11-05

**Authors:** Ioannis Theologidis, Ivo M Chelo, Christine Goy, Henrique Teotónio

**Affiliations:** Instituto Gulbenkian de Ciência, Apartado 14, Oeiras, P-2781-901 Portugal; Benaki Phytopathological Institute, Stefanou Delta 8, Kifissia, 14561 Greece; Leibniz Research Institute for Environmental Medicine, Düsseldorf, 40225 Germany; École Normale Supérieure, Institut de Biologie de l’ENS (IBENS), Inserm U1024, CNRS UMR 8197, Paris, F-75005 France

**Keywords:** Evolutionary transition, Selfing, Androdioecy, Fitness, Experimental evolution, *Caenorhabditis elegans*, *fog-2*, *xol-1*, NaCl

## Abstract

**Background:**

Evolutionary transitions from outcrossing between individuals to selfing are partly responsible for the great diversity of animal and plant reproduction systems. The hypothesis of ‘reproductive assurance’ suggests that transitions to selfing occur because selfers that are able to reproduce on their own ensure the persistence of populations in environments where mates or pollination agents are unavailable. Here we test this hypothesis by performing experimental evolution in *Caenorhabditis elegans*.

**Results:**

We show that self-compatible hermaphrodites provide reproductive assurance to a male-female population facing a novel environment where outcrossing is limiting. Invasions of hermaphrodites in male-female populations, and subsequent experimental evolution in the novel environment, led to successful transitions to selfing and adaptation. Adaptation was not due to the loss of males during transitions, as shown by evolution experiments in exclusively hermaphroditic populations and in male-hermaphrodite populations. Instead, adaptation was due to the displacement of females by hermaphrodites. Genotyping of single-nucleotide polymorphisms further indicated that the observed evolution of selfing rates was not due to selection of standing genetic diversity. Finally, numerical modelling and evolution experiments in male-female populations demonstrate that the improvement of male fitness components may diminish the opportunity for reproductive assurance.

**Conclusions:**

Our findings support the hypothesis that reproductive assurance can drive the transition from outcrossing to selfing, and further suggest that the success of transitions to selfing hinges on adaptation of obligate outcrossing populations to the environment where outcrossing was once a limiting factor.

**Electronic supplementary material:**

The online version of this article (doi:10.1186/s12915-014-0093-1) contains supplementary material, which is available to authorized users.

## Background

Evolutionary transitions from outcrossing, where individuals mate and cross-fertilize each other, to self-fertilization (henceforth, selfing) are in part responsible for the great diversity of animal and plant reproduction systems [1-6]. Transitions to selfing have repeatedly occurred during evolution even though this breeding mode usually leads to inbreeding depression [1,7], restricts the generation of potentially adaptive genetic diversity [8-10] and may result in an increased probability of extinction [11-15]. Countering these and other shortcomings of selfing, by being able to reproduce on their own, autonomous selfers may ensure the persistence of populations facing an environment where mates or pollination agents are unavailable [1,16,17]. ‘Reproductive assurance’ could thus provide an explanation for transitions to selfing.

Despite the considerable efforts trying to demonstrate the hypothesis of reproductive assurance, empirical evidence is mixed [18-21], and whether individual selection can drive the transition to selfing has not been subject to direct experimental tests; but see [22-24]. This is partly because hermaphroditism and selfing occur in multiple forms, not all of which clearly assure population persistence in environments with limited opportunity for outcrossing [17,25-28]. Perhaps more significantly, it has been difficult to disentangle individual selection among selfers and outcrossers from other processes that may also result in the evolution of increased selfing rates.

When mutation to self-compatibility is non-limiting [29-31], increased selfing rates can evolve due to the numerical transmission advantage that a selfing allele has over an outcrossing allele, if selfers are also able to outcross with each other [20,32]. This process mitigates the ‘cost of meiosis’ that results from outcrossed progeny inheriting only half the gene complement of their parents when compared to selfed progeny [33,34]. Second, and depending on the genetics of sex determination, increased selfing rates can result from the elimination of a ‘cost of males’, as male progeny cannot reproduce on their own but consume resources that may be critical for future generations [34,35]. Third, increased selfing rates can evolve as a correlated response to density dependent selection for higher dispersal to and/or colonization ability of novel habitats [36-39]. This is the case, for example, in the evolution of selfing in metapopulations, where density dependent selection among selfing and outcrossing demes experiencing frequent extinction may be confounded with individual selection [38,40]. Lastly, it is largely unknown how selection of standing genetic diversity specific to breeding mode [41,42], such as the purging of deleterious recessive alleles [43-45] or the maintenance of coevolved sets of loci [46-49], relate to reproductive assurance and may feedback on the evolution of selfing rates.

If transitions to selfing result from individual selection among selfers and outcrossers in an environment where outcrossing is restricted, then successful transitions should lead to adaptation [17,28]. Here, we perform experimental evolution in populations of *Caenorhabditis elegans* nematodes to test this prediction of the reproductive assurance hypothesis.

*C. elegans* is an androdioecious nematode because males co-occur with self-compatible hermaphrodites [50]. Since hermaphrodites cannot outcross each other, *C. elegans* lineages do not suffer from a cost of meiosis although they suffer a fitness cost when segregating males [51-53]. *C. elegans* sex determination is chromosomal, such that hermaphrodites are XX and males are XØ as a result of the non-disjunction of the X-chromosome during hermaphrodite gametogenesis [54-56]. In natural populations, males primarily arise from X-chromosome non-disjunction [56,57]. In recent years, however, it has been demonstrated in the laboratory that males can be maintained for relatively long periods at intermediate to high frequencies, from 10% to 50% for up to 100 generations, under several challenging circumstances, such as the presence of pathogens or genetic loads [55,58-63].

**Figure 1:**
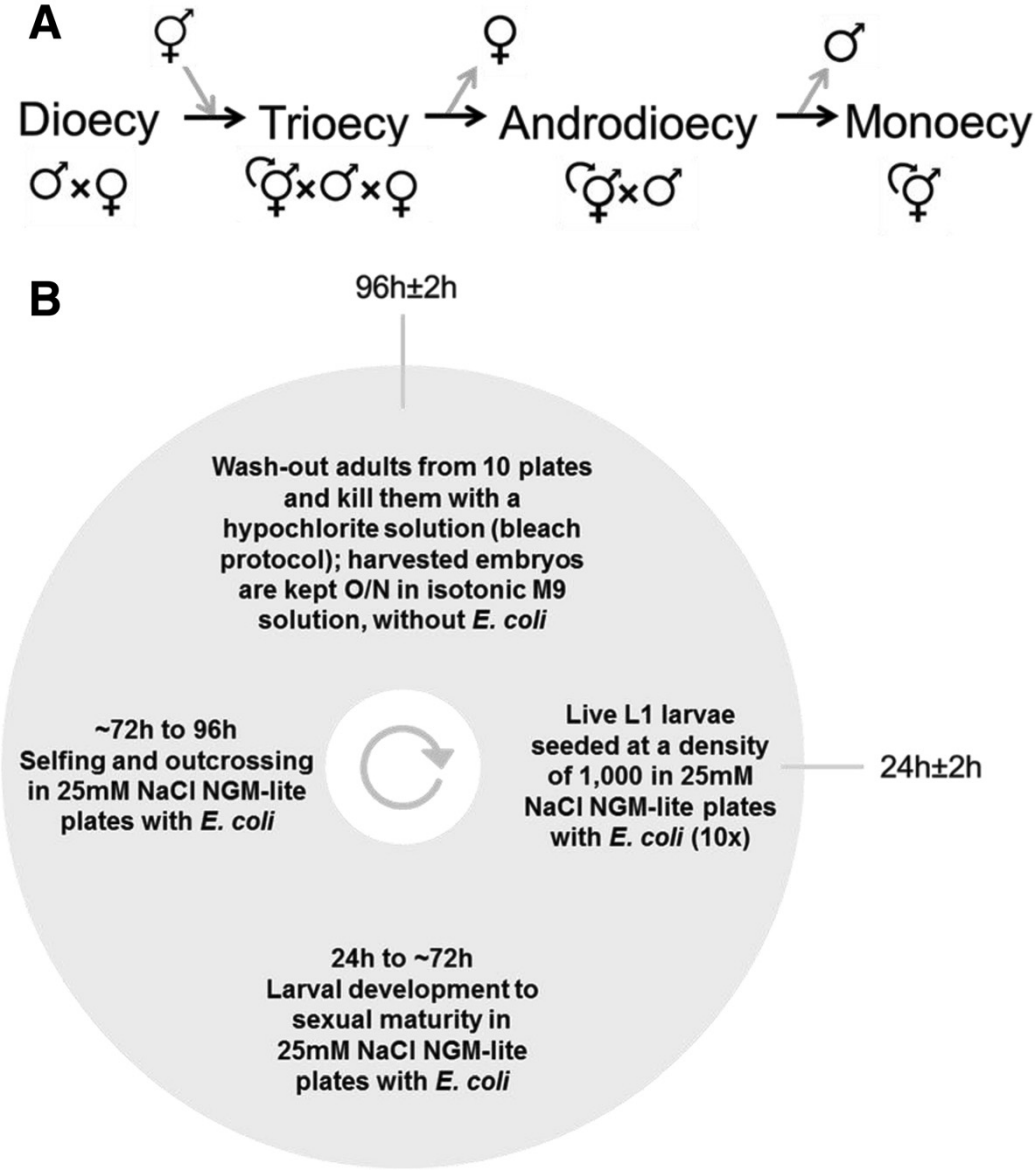
**Evolutionary transitions to selfing in** ***Caenorhabditis*** **nematodes and the life cycle of a laboratory-adapted** ***C. elegans*** **population. (A)** Transitions in reproductive systems and breeding modes that three *Caenorhabditis* nematode species may have undergone in their history (black arrows). Like most extant *Caenorhabditis* species, the ancestral populations had a dioecious reproduction system until the appearance of a mutant female turned self-compatible hermaphrodite (grey arrow), which transformed them into trioecious populations. Females and hermaphrodites can only mate with males. Successful transitions to selfing imply invasion of hermaphrodites, loss of females and the maintenance of mixed selfing and outcrossing under androdioecy, or further loss of males and exclusive hermaphroditism (which is here defined as monoecy since we ignore mutation in sex determination). **(B)** The four-day discrete non-overlapping life cycle of a lab-adapted population, ancestral to all populations here constructed, experimentally evolved and characterised. With the exception of NaCl, the life cycle and population census sizes were the same as the experimental evolution here reported. Grey lines indicate the time points of experimental manipulation. Between 24 hours of the life cycle, when the first larval staged L1 individuals are seeded in solid NGM-lite plates, until 96 hours, when adults reproduce and give rise to the embryos that will constitute the next generation, NaCl concentrations in the media were 25 mM. Increased NaCl concentrations to 305 mM is here used as the novel environment.

Within the *Caenorhabditis* clade, androdioecy derived from male-female dioecy at least three times [64,65]. Theoretical studies have supported the following model for these transitions [51-53,66] (see Figure 1A). A mutant female turned self-compatible hermaphrodite invaded the ancestral dioecious population because the probability of females failing to reproduce was higher than that of hermaphrodites in environments with limited outcrossing. At this stage, individual hermaphrodites were selected owing to the reproductive assurance they afforded the dioecious population, despite inbreeding depression and despite population density dynamics; but see [67,68]. Also during this stage, fitness components in selfing hermaphrodites traded-off with male and female fitness components, but the maintenance of trioecy (with the three sexes) was unlikely to occur and hermaphrodites eventually displaced females. This is because females would need to evolve in a relatively short period at least double the reproductive output of hermaphrodites in order to be maintained; see [68,69] for further discussion. After, maintenance of selfing and outcrossing under androdioecy would have been possible if males sired at least twice the number of selfed progeny, thus offsetting the cost of males. But effective monoecy and exclusive hermaphroditism was the probable end result of the transition, as in most extant populations males commonly appear through the non-disjunction of X-chromosome during hermaphrodite gametogenesis.

Following this framework, we conducted invasions of wild-type hermaphrodites in genetically transformed dioecious populations to test for transitions to selfing and subsequent adaptation in a novel environment where hermaphrodites provide reproductive assurance. To quantify the extent of selection on hermaphrodites, we submitted genetically transformed monoecious populations to experimental evolution in the same environment, and experimental evolution of androdioecious populations was also performed to determine whether the loss of males was sufficient to explain adaptation. Genotyping of single-nucleotide polymorphisms across one autosome was done to test if selection of standing genetic diversity could influence the evolution of selfing rates during transitions. Finally, we numerically modelled the evolution of fitness components expressed under outcrossing, during the trioecious phase of the transition, and asked if the experimental evolution of increased male performance under dioecy could determine the opportunity for reproductive assurance.

## Results and discussion

### Outcrossing is limited at high NaCl concentrations

Fortuitous observations suggested that NaCl influenced the selfing rates of our laboratory adapted androdioecious population. This population had been cultured for 140 generations under discrete non-overlapping four-day life cycles, at constant and spatially homogenous densities, while keeping stable selfing rates (Figure 1B) [55,70]. NaCl concentrations in the culture media plates during experimental evolution were 25 mM between the first larval stage (L1) to adult reproduction (between 24 hours and 96 hours of the life cycle). Unlike natural populations, this lab-adapted population has abundant genetic diversity since it was originally derived from a cross of several natural isolates and was subsequently maintained at census sizes of N = 10^4^ and effective sizes of N_e_ approximately = 10^3^ [71].

**Figure 2:**
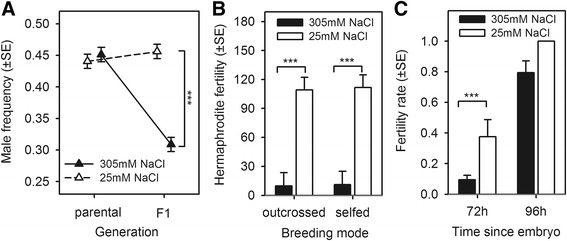
**Outcrossing is limited at high NaCl concentrations. (A)** Development from the L1 larval stage (24 ± 2 hours) to the time of reproduction (96 ± 2 hours) in varying NaCl concentrations did not affect male frequencies in the lab-adapted population (parental generation, P). Sex ratios in the following F1 generation were affected, however, with a high NaCl concentration reducing population male frequencies when compared to a low NaCl concentration (generalised linear mixed effects model (GLMM): z-ratio test for a significant difference at F1 among salt environments: |z| =12.2, *P*-value <0.001, residual d.f. =91; see Methods for justification of statistical modelling]. **(B)** Despite the large impact of high NaCl concentrations in hermaphrodite fertility from 72 ± 2 hours to 96 ± 2 hours (GLMM: main effect NaCl |z| =27.5, *P*-value <0.001, residual d.f. =123), outcrossed hermaphrodites had the same fertility as selfed hermaphrodites in high or low salt (*post hoc* Tukey comparisons at 305 mM NaCl: |z| =0, *P*-value =0.99; at 25 mM NaCl |z| =0.3, *P*-value =0.78). **(C)** Development to maturity since the L1 larval stage was retarded at 305 mM NaCl since there was a reduced proportion of fertile hermaphrodites in the lab-adapted population at this concentration (generalised linear model (GLM): main effect NaCl |z| =2.97, *P*-value =0.003, residual d.f. =6). After 72 ± 2 hours of development in 305 mM NaCl only 10% of the hermaphrodites were fertile when compared to the 40% in 25 mM NaCl (|z| =3, *P*-value =0.003). However, after 96 ± 2 hours differences among NaCl environments were no longer significant (|z| =0.001, *P*-value =0.99). For all panels, triangles, bars and standard errors depict ordinary least-square estimates; ***indicates *P*-values <0.001. d.f., degrees of freedom.

In the lab-adapted population, we measured male proportions at 96 hours of the life cycle in two consecutive generations of culture at 305 mM NaCl (henceforth, high salt) or at the concentration to which they were adapted, 25 mM NaCl (henceforth, low salt). In the first generation of the assay, salt did not affect male proportions, a result indicating that the relative survivorship of males and hermaphrodites from the L1 larval stage to reproductive maturity is independent of salt (Figure 2A; all statistical significance results in the figure legends). In the second generation, however, adult male numbers were lower in high salt when compared to low salt.

Selfing rates, calculated as one minus twice the male proportions at the second generation [52], were 0.09 ± 0.01SE in low salt and 0.38 ± 0.01SE in high salt. These estimates assume that there were no mixed broods with selfed and outcrossed progeny, which is likely since once hermaphrodites mate with males, male sperm outcompetes self-sperm for fertilization [72]. Further assays with increasing salt concentrations revealed that selfing rates exhibited a significant increase only above 275 mM (see Additional file 1: Figure S1), and that there were no maternal effects of salt on embryo-to-adult hermaphrodite survivorship (see Additional file 1: Figure S2). Together these findings show that high salt limits outcrossing and favours selfing.

### Delayed hermaphrodite developmental time underlies limited outcrossing

Differential survivorship of males and hermaphrodites does not seem to explain limited outcrossing in high salt, so we tested an alternative explanation—that hermaphrodite fertility is lower under outcrossing than selfing. (Fertility hereafter defined as fecundity, the number of embryos laid during the 24 hours preceding the time of reproduction and population passage, and survivorship until adulthood). To test for hermaphrodite fertility, independently of male effects, hermaphrodites from the lab-adapted population were outcrossed to males from an unrelated green fluorescent protein (GFP) inbred tester. The resulting hermaphrodite progeny was then scored for fertility under outcrossing, when hermaphrodites were again mated to the unrelated GFP tester males, and under selfing (see Methods). Results from this assay showed that despite the large impact of salt on fertility, outcrossed hermaphrodites had the same fertility as selfed hermaphrodites in high or low salt (Figure 2B).

Several other trait changes can explain limited outcrossing in high salt. High salt concentrations are known to affect hermaphrodite survival, body size and chemotaxis [73,74], traits that in turn can determine impaired male mating behaviour or hermaphrodite resistance to mating [75-77]. However, there may be simply a reduced time window for outcrossing if hermaphrodites take longer to reach maturity in high salt than low salt [78,79]. To confirm this, we scored the proportion of fertile hermaphrodites in the lab-adapted population at 72 hours and 96 hours of the life cycle (Figure 1B). We observed that over both periods, high salt reduced fertility rates, revealing delayed developmental time to maturity (Figure 2C). Specifically, at 72 hours, almost 40% of the hermaphrodites were fertile after development in low salt but only approximately 10% were fertile in high salt. At 96 hours, all hermaphrodites were fertile in low salt and about 80% were fertile in high salt, although this difference was not statistically significant.

### Hermaphrodites provide reproductive assurance to a dioecious population

With limited outcrossing in high salt selfed hermaphrodites could provide reproductive assurance to a dioecious population exposed to this environment. To construct a dioecious population, we recurrently introgressed the self-sperm knockout allele, *fog-2(q71)*, in the androdioecious lab-adapted population (see Methods). *fog-2(q71)* is a recessive autosomal allele that transforms hermaphrodites when homozygous [80]. In low salt, *fog-2(q71)* is inconsequential to male or female reproductive success under the life-cycle we employed [55,81] (Figure 1).

**Figure 3:**
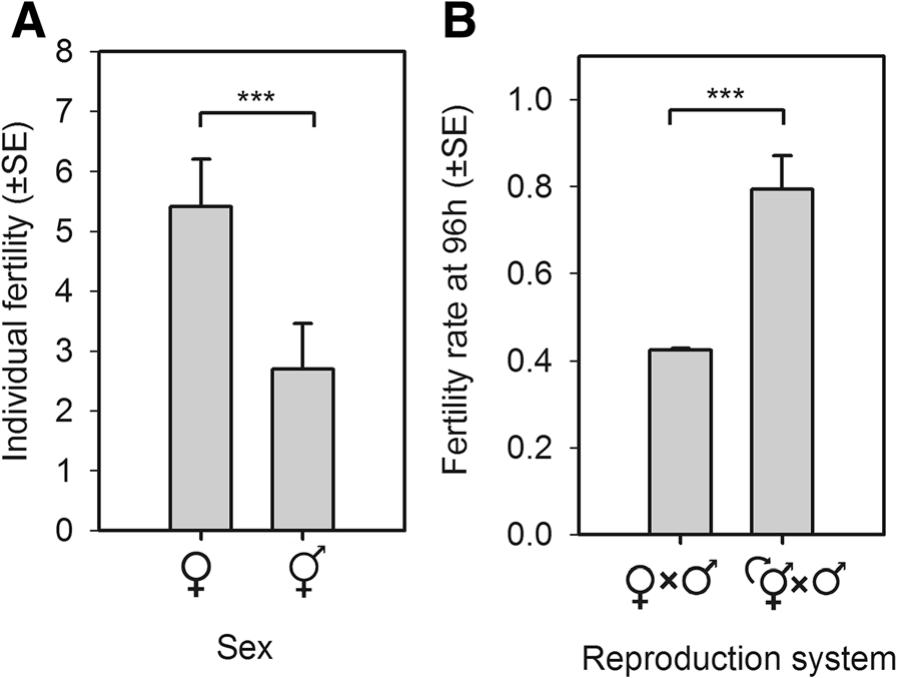
**Reproductive assurance in a dioecious population. (A)** In high salt, the fertility of females from the genetically-transformed dioecious population was higher than the fertility of hermaphrodites from the lab-adapted population (GLM: |z| =5, *P*-value <0.001, residual d.f. =55). **(B)** In high salt and at the time of population passage, the fertility rate of the genetically transformed dioecious population was lower than that of the lab-adapted androdioecious population (GLM: |z| =4.25, *P*-value <0.001, residual d.f. =3). For all panels, bars and errors show ordinary least-square estimates; *** indicates *P*-values <0.001. d.f., degrees of freedom; GLM, generalized linear model.

To test for reproductive assurance we first showed that females from the dioecious population do not have reduced fertility when compared to wild-type hermaphrodites. We characterised the fertility of hermaphrodites and females when mated with males from the dioecious population, for the 24 hours preceding population passage and after development to maturity since the L1 larval stage in high salt (we did not differentiate the progeny of selfed and outcrossed *hermaphrodites*; see Methods). This assay indicated that females had higher fertility than hermaphrodites when in high salt conditions (Figure 3A).

If females have higher fertility than hermaphrodites, then hermaphrodites can provide reproductive assurance to the dioecious population only when a higher proportion of females fails to reproduce than hermaphrodites. To show the possibility for reproductive assurance, we measured the fertility rates of the dioecious population in high salt conditions following the same protocol as above for the lab-adapted androdioecious population (see Methods). Results from this assay indicated that at 96 hours of the life cycle, the time of reproduction and population passaging under our imposed life-cycle (Figure 1), about 40% of females were fertile, a significantly lower proportion than the approximately 80% of hermaphrodites that were fertile in the androdioecious population (Figure 3B).

It is unclear if the higher individual female fertility than individual hermaphrodite fertility compensates for the lower fertility rate of the dioecious population than the androdioecious population in high salt. The individual fertility assays were done under male density, population density and life cycle timing conditions that were not those of our standard population maintenance protocol, but that were followed in the population fertility rate assay (see Figure 1 and Methods for further details). We, therefore, conclude that selfed hermaphrodites provide reproductive assurance to the dioecious population when in high salt since a higher proportion of females than hermaphrodites failed to reproduce at the time of population passaging.

### Experimental transitions to selfing

An intermediate stage in the construction of the dioecious population involved the creation of a trioecious population, where the wild type *fog-2* selfing allele was at a frequency of 2.6 × 10^-2^ and, therefore, where hermaphrodites were at an expected frequency of 6.8 × 10^-4^ (see Methods). This male-female-hermaphrodite population should mimic the ancestral dioecious reproduction system of *Caenorhabditis* species, soon after the appearance of a mutant female turned self-compatible hermaphrodite that has not been lost by genetic drift [70] (Figure 1). Using replicates of this population we performed evolution experiments in three different salt regimes to test whether reproductive assurance results in transitions to selfing. Importantly, except for salt concentrations and sex ratios, all environmental conditions and population census sizes during experimental evolution were the same as those employed during previous laboratory adaptation (Figure 1B).

**Figure 4:**
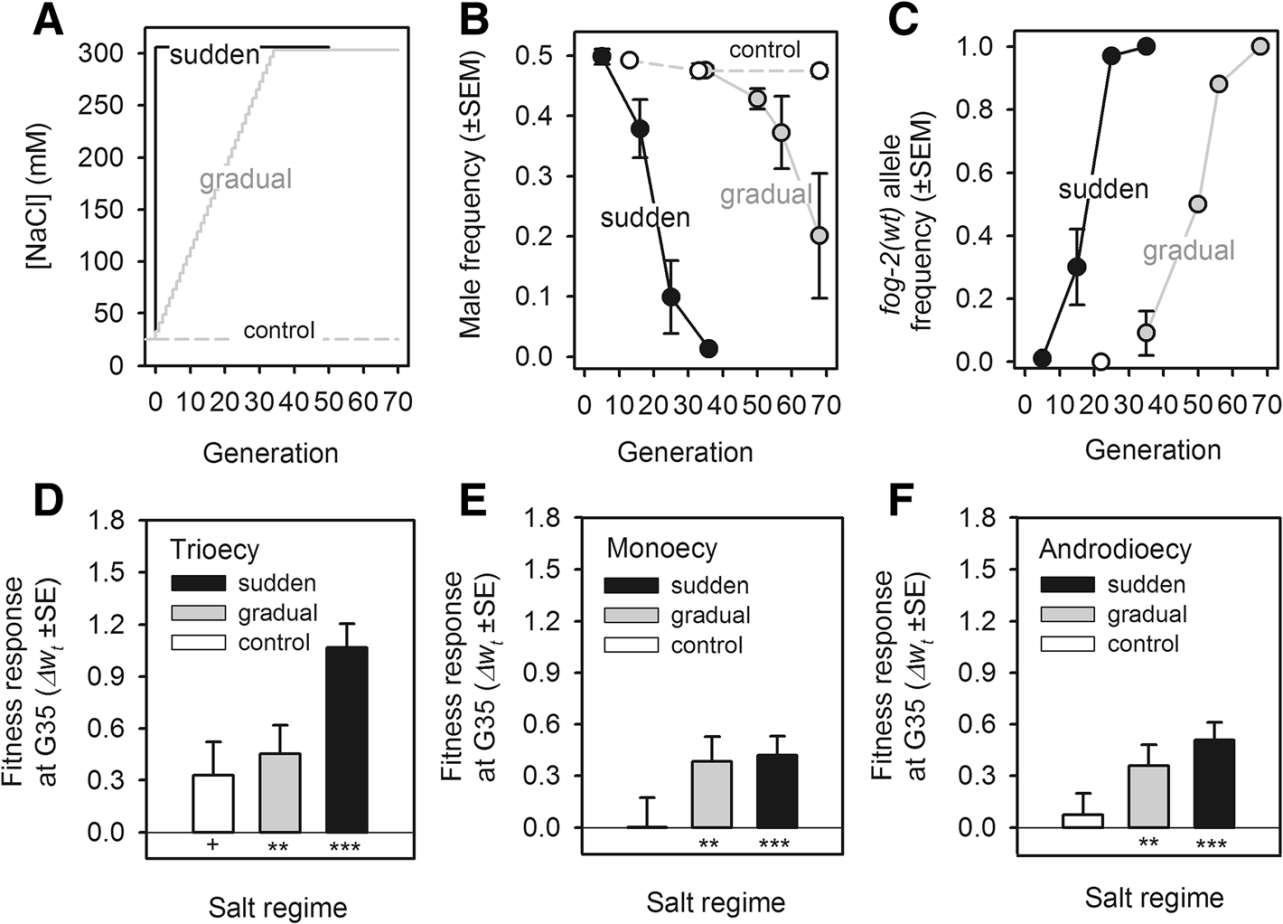
**Experimental transitions to selfing result in adaptation. (A)** Experimental design. **(B)** Trioecious populations lost males when cultured at 305 mM NaCl. **(C)** Dynamics of the selfing allele. Transition fitness was significantly different from zero in the sudden and gradual regimes (both analysis of variance (ANOVA) *P*-values <0.001, residual d.f. =10). B and C, circles are the observed mean values of replicate populations with error bars one standard mean error among them. **(D)** Fitness response of trioecious populations after 35 generations of evolution (*Δw*_*t*_). A linear mixed effects model (LMM) shows that populations from all regimes adapted to 305 mM (control test against the zero intercept: |z| =1.76, *P*-value =0.09; gradual: |z| =2.7, *P*-value =0.007; sudden: |z| =7.8, *P*-value <0.001; residual d.f. =65). *Post hoc* Tukey tests show that sudden populations had a higher fitness response than either gradual or control populations (sudden versus gradual t_17_ = 2.5, *P* =0.05; sudden versus control t_17_ = 2.74, *P*-value =0.04; gradual versus control t_17_ = 0.4, *P*-value =0.9). **(E)** As panel D, but for monoecious populations. LMM shows a fitness response in 305 mM NaCl by G35 in sudden and gradual populations (control: |z| =0.28, *P*-value =0.98; gradual: |z| =2.6, *P*-value =0.009; sudden: |z| =3.8, *P*-value <0.001; residual d.f. =69; no *post hoc* differences between regimes). **(F),** as panels D and E, but for androdioecious populations. LMM shows a fitness response in 305 mM NaCl by G35 in sudden and gradual populations (control: |z| =0.64, P-value =0.52; gradual: |z| =3, *P*-value =0.003; sudden: |z| =4.9, *P*-value <0.001; residual d.f. =55; no *post hoc* differences between regimes). For panels D, E and F, bars and errors show LMM estimates. +, *, **, *** indicate, respectively, *P*-values of <0.1, <0.05, <0.01 and <0.001. d.f., degrees of freedom.

In the ‘sudden’ regime, populations were immediately introduced to, and subsequently cultured at, 305 mM NaCl between 24 hours and 96 hours of the life cycle (Figure 4A; Additional file 1: Table S1 for replication structure). Here we expected selfing rates to greatly increase during the first few generations of experimental evolution. In the ‘gradual’ regime, populations were cultured at linearly increasing NaCl concentrations until 305 mM at generation 35, and were then maintained in high salt. Here we expected males and females to persist at high frequencies until 275 mM NaCl since only at or above this salt concentration is outcrossing limited (see Additional file 1: Figure S1). In the ‘control’ regime, populations were cultured in the same low salt conditions as the lab-adapted population. Populations from this regime allowed us to control for any evolutionary changes associated with the introgression of the *fog-2(q71)* allele in the lab-adapted population, and the new sex ratio condition, despite salt.

We measured both male and *fog-2* genotype frequencies to describe transitions to selfing during experimental evolution. As expected, male numbers in the sudden regime rapidly declined, reaching negligible levels by generation 35 (Figure 4B). Following the expected dynamics, the *fog-2(wt)* selfing allele increased in frequency and reached fixation by generation 35 (Figure 4C). Estimating fitness of selfing as the haploid coefficient describing the deterministic frequency change of the selfing allele relative to the resident *fog-2(q71)* allele during experimental evolution [70,82] results in a value of s =0.32 ± 0.03SE (see Methods; we call *s* the ‘transition fitness’).

In the gradual regime, male numbers began to decline only after generation 35 and at the final generation of culture (generation 68) males were still present at intermediate frequencies. The selfing allele reached fixation in the gradual populations after approximately 30 generations in high salt, mirroring the observations made in the sudden populations. In the gradual populations, the transition fitness of the selfing allele was of s =0.22 ± 0.02SE, a significantly lower value than the corresponding value in the sudden populations.

In the control populations, the male and the selfing allele frequencies remained at ancestral levels during experimental evolution.

Despite the higher fertility of outcrossed females compared to hermaphrodites (shown in Figure 3A), hermaphrodites were able to invade and fix. Experimental transitions to selfing were thus successful when populations were confronted with a high salt environment, from dioecy to monoecy, in the case of the sudden populations, and from dioecy to androdioecy, in the case of the gradual populations (see Figure 1B). Control populations in turn maintained effective dioecy during experimental evolution.

### Transitions to selfing result in adaptation

The key prediction of the hypothesis of selection for reproductive assurance is that successful transitions to selfing result in adaptation to the environment where outcrossing is a limiting factor [17,28,51]. In contrast, populations that do not transit to selfing should not adapt through selection for reproductive assurance. They could adapt, however, to their new male-female sex ratio condition that the lab-adapted population had not experienced during its history. Control populations, like the gradual populations, could only adapt to their new sex ratio condition that the lab-adapted population had not experienced during its history.

We measured fitness of the evolved populations at generation 35 in parallel with that of the lab-adapted ancestral androdioecious population under high salt conditions. Fitness was estimated with competition assays between the experimental populations and a GFP androdioecious tester population which segregated 5% of males but was otherwise similar to the lab-adapted ancestral population (see Methods; note that this GFP tester population is not the GFP tester inbred strain used in the individual fertility assay reported above). Adaptation was then equated with the changes in the haploid coefficient describing the deterministic frequency change of the wild type allele relative to the GFP tester allele in the evolved populations relative to the ancestral populations [70,83] (see Methods and Additional file 1: Figures S3,S4 for details on the assay design and data quality control).

Populations from all regimes increased their fitness in high salt after 35 generations of experimental evolution (Figure 4D). However, sudden populations increased their fitness to a greater extent than the gradual populations. Control populations also increased their fitness and to a similar extent of the gradual populations. Populations that had a successful transition to selfing, therefore, had higher adaptive rates than those that did not transit to selfing.

Fitness responses in the gradual populations must have been due to selection on males and females, as they maintained effective dioecy until generation 35. Further, selection on males and females appears to have been independent of salt conditions since the control populations showed similar responses to those of the gradual populations, and they also maintained effective dioecy until generation 35. Following a similar reasoning, selection on males and females could also have contributed to the adaptive responses of the sudden populations, as males and females were maintained at relatively high frequencies until generation 30 (see Figure 4B,C). If so, alleles selected in this manner became associated with selfing in order for fitness differences to be detected by generation 35, when there were only hermaphrodites. Together with selection on hermaphrodites, selection on males and females may have thus been responsible for higher adaptive rates in the sudden populations than the gradual or control populations.

### Selection on hermaphrodites and adaptation

We have assumed so far that the sudden and gradual salt regimes imposed the same selection gradients on the relevant fitness traits [84]. It could be, however, that selection gradients under the gradual and control regimes were less steep than in the sudden regime, that is, selection on the relevant traits was weaker, thus explaining the relatively slower adaptive rates of gradual and control populations when compared to the sudden populations. To test this assumption for hermaphrodite traits, we conducted experimental evolution with (hermaphroditic) monoecious populations and subjected them to the sudden, gradual and control salt regimes. With the exception of those traits that are specific to the interactions between the sexes, similar traits should be expressed by hermaphrodites in the monoecious and the trioecious populations that underwent experimental evolution [81,85].

To create the ancestral monoecious population, the male-killing mutant allele, *xol-1(tm3055)*, was recurrently introgressed in the lab-adapted population (see Methods; Additional file 1: Table S1). Following the same protocol as above, we characterised adaptation to high salt with competition assays. After 35 generations of experimental evolution, monoecious populations had similar fitness responses when maintained in the sudden or gradual regime, while there was no fitness response in control populations (Figure 4E). Given these results, we can conclude that selection gradients on hermaphrodite traits expressed under selfing were of similar magnitude under the gradual and the sudden regimes. Higher adaptive rates in the sudden regime were likely not due to selection differences to other regimes.

### Transitions to selfing and the cost of males

To address if fitness responses during the transitions to selfing could be explained by the loss of males and, consequently, an increase in the number of breeding individuals [34], we did experimental evolution under androdioecy, also in the three different salt regimes. Since there are no females under androdioecy, hermaphrodites do not reproductively guarantee population persistence. But as in the trioecious populations, males may impose an up to two-fold fitness reduction, that is, a cost of males, as sex ratio segregation is the same whether males outcross with females or with hermaphrodites [55] and both trioecious and androdioecious populations started experimental evolution with similar sex ratios (see also Additional file 1: Figure S5).

As with the trioecious populations, androdioecious populations of both salt regimes increased their selfing rates immediately upon culture in high salt (see Additional file 1: Figure S6). The gradual populations, in particular, maintained a high frequency of males until generation 35, at which point male numbers declined. Using the same competition assays as above to describe adaptation to high salt, we found that, unlike in the trioecious populations, both the sudden and the gradual androdioecious populations had similar fitness responses after 35 generations of experimental evolution (Figure 4F). Control androdioecious populations showed no sign of adaptation.

The fitness responses under androdioecy show that compromised adaptive rates in the gradual trioecious populations, which until generation 35 remained effectively dioecious, cannot be explained by the loss of males and, therefore, by the existence of a cost of males. The loss of males in the sudden trioecious populations, which by generation 35 had transitioned to selfing, did not facilitate adaptation. Instead, we can conclude that facilitated adaptation in the sudden trioecious populations was due to displacement of females by hermaphrodites. In other words, transitions to selfing occurred because hermaphrodites provided reproductive assurance to the ancestral (effectively) dioecious population.

### Selection of standing genetic diversity

Selfing structures standing genetic diversity in complex ways that could have contributed to the evolution of selfing during the experimental transitions, thus confounding the role of reproductive assurance [42,71,86,87]. One such possibility is that, once hermaphrodites became relatively common, increased segregation of homozygotes would allow efficient purging of deleterious recessive alleles, which would lead to increased selfing rates [43-45]. Another possibility is that coevolved sets of loci present in the lab-adapted population were, by chance, adaptive in the novel high salt environment. If so, once hermaphrodites were relatively common, increased selfing would generate high linkage disequilibrium and reduce effective population recombination rates [8], allowing maintenance of undisrupted coevolved sets of loci, which would in turn favour increased selfing rates [46,47,49,71].

One can thus ask whether selection of standing genetic diversity determined adaptation independently of selfing. If that was the case, then population genetic processes can explain transitions to selfing despite selection for reproductive assurance. To address this question, we measured the levels and structure of genetic diversity in populations from all reproduction systems (trioecy, monoecy, androdioecy) and all regimes (sudden, gradual, control) at a generation that was late enough during the focal trioecious experiment for hermaphrodites to be common but early enough during the experiments for standing genetic diversity to influence adaptation at generation 35. Genotyping was done at 58 single-nucleotide polymorphisms (SNPs) across chromosome IV at generation 22 of experimental evolution (see Additional file 2). These SNPs should mostly behave as neutral markers linked to selected alleles, and if there was selection on deleterious recessive alleles or on coevolved sets of loci, we expected the SNP homozygosity or linkage disequilibrium at generation 22 to correlate with the fitness responses observed at generation 35.

**Figure 5:**
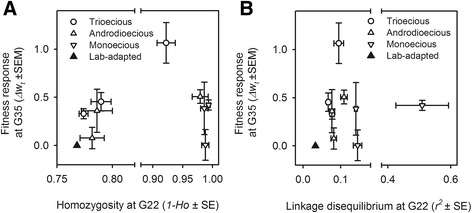
**Selection of standing genetic diversity does not explain adaptation.** Bi-plots of the fitness responses at generation 35 of experimental evolution (*Δw*_*t*_; shown in Figure 4) with the homozygosity **(A)** or pairwise linkage disequilibrium **(B)** of single nucleotide polymorphisms genotyped at generation 22 (see Methods). Axes are truncated for clarity. Symbols show the observed mean and the standard error of the mean among replicate populations of the three reproduction systems, as well as the mean of the lab-adapted androdioecious population, ancestral to them all. Analysis of this data is presented in Table 1.

Analysis of SNP genotypes revealed that, by itself, selfing greatly increased homozygosity and linkage disequilibrium (Figure 5; see Methods for details on genotyping and diversity statistics). This was more evident when comparing the monoecious populations to the ancestral lab-adapted population. However, when the effects of reproductive system on the fitness responses are taken into account and, therefore, the effects of selfing on structuring genetic diversity, neither the amount of homozygosity nor linkage disequilibrium explain the fitness differences between evolved and ancestral populations (Table 1). We can thus conclude that selection of standing genetic diversity did not greatly influence the evolution of selfing during the experimental transitions.

**Table 1:** **Selection of standing genetic diversity does not explain adaptation**

**Independent variable**	**d.f.**	**SS**	**MS**	**F-value**	***P*** **-value**
Homozygosity	1	0.069	0.069	0.569	0.456
Reproduction system	2	2.349	1.175	9.643	0.001
Error	33	4.019	0.122	-	-
Linkage disequilibrium	1	0.034	0.034	0.209	0.650
Reproduction system	2	1.391	0.0696	4.309	0.022
Error	31	5.005	0.161	-	-

Table shows the two analyses of covariance done to test if the fitness responses at generation 35 (*Δw*_*t*_) are explained by SNP homozygosity (*1-Ho*) or pairwise SNP linkage disequilibrium (*r* ^*2*^) at generation 22 (see also Figure 5 and Methods for details on statistical models and calculations of genetic diversity). In each model, reproduction system (Trioecy, Monoecy, Androdioecy) is treated as the fixed independent variable. SS and MS stand for Sum-of-Squares and Mean-of-Squares, respectively. Error degrees of freedom (d.f.) for the model testing the effects of linkage disequilibrium is 31 because two monoecious populations from the sudden regime had such low SNP diversity that *r* ^*2*^ could not be calculated (see Additional file 1: Figure S4).

### Modelling the evolution of the fitness components of outcrossing

During the transitions to selfing in the sudden regime, hermaphrodites displaced females and males soon after 30 generations of evolution in high salt (Figure 4B,C). In contrast, males were still present at relatively high frequencies at the end of experimental evolution in the gradual regime. Furthermore, transition fitness of the selfing allele was higher in the sudden than in the gradual regime. A reasonable hypothesis to explain these differences between salt regimes is that the evolution of traits expressed by the three sexes under outcrossing contributed to evolution of selfing rates during the transient trioecious phase, the more so in the gradual than in the sudden regime [51,81,85]. To illustrate the potential evolution of the fitness components of outcrossing during transitions (‘outcross-fitness’ for short), we asked how they could explain the observed frequency dynamics of the *fog-2(wt)* selfing allele.

Barring mutation in sex determination [29,55,88,89], maintenance of androdioecy in *C. elegans* depends on the male reproductive success relative to the hermaphrodite reproductive success (α), on the embryo to maturity survivorship difference between hermaphrodites and males (σ), on the proportion of non-cross-fertilised oocytes that are self-fertilised (β) and on inbreeding depression for fitness components (δ). *C. elegans* hermaphrodites are protandrous in that they first produce sperm and then irreversibly switch to oogenesis upon reproductive maturation [78]. If these parameters do not depend on male or hermaphrodite density, then maintenance of androdioecy is possible when: α(1-σ) >2β(1-δ), where ‘2’ accounts for the two-fold cost of males; see [52,67,90] for details and further discussion.

We revised this androdioecious model to numerically simulate the experimental transitions to selfing by accounting for females in the segregation of *fog-2* genotypes (see Methods; the expected reproduction table under trioecy is presented in Additional file 1: Table S2). For simplicity, we assumed no mixed broods so that when selfed each hermaphrodite was assigned 2β =2. Conversely, unmated females could not reproduce and were assigned 2β =0. Since, in the lab-adapted population, we did not find survivorship differences between hermaphrodites and males exposed to varying salt concentrations, we set σ =0 (see Figure 2A and Additional file 1: Figure S1). We also considered that δ =0 since we found no signs of inbreeding depression in the lab-adapted population (see Figure 2B) and this parameter should not, in most cases, influence transitions to selfing, see for example [67]. Finally, we defined *α* as the parameter representing all fitness components expressed under outcrossing rather than specifically modelling female survivorship. With these settings, androdioecy is maintained when the magnitude of outcross-fitness offsets the two-fold cost of males: α >2.

**Figure 6:**
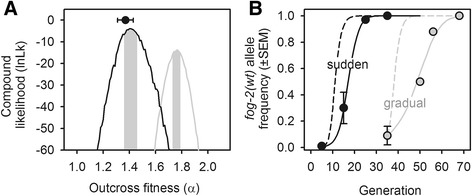
**Expected** ***fog-2*** **genotype frequency dynamics during transitions. (A)** Compound likelihood curves of the outcross-fitness values (*α*) that in simulations of trioecious experimental evolution can explain the observed *fog-2(wt)* allele frequency dynamics in the sudden population (black) and in the gradual populations (grey). Grey bars indicate the credible interval of -2lnLk around the maximum-likelihood (ML) estimate. Circle shows the expected outcross-fitness component value (α) with one SE in the lab-adapted population, using the data from Figure 2A (see Methods). **(B)** Deterministic *fog-2(wt)* allele trajectories expected with the ML α estimates (lines). Circles and error bars are the observed trajectories (as in Figure 4C). Dashed lines illustrate the deterministic *fog-2(wt)* frequency dynamics if there is no cost of males as modelled with α =1. See also Additional file 1: Figure S8 for the sex ratio experimental evolution expectations. Simulations assumed random mating and selfing and no sex segregation distortion (see Additional file 1: Table S2).

Individual-based Monte Carlo simulations were done following the experimental evolution life cycle and census sizes in order to obtain the expected *fog-2* genotype frequency trajectories under several values of α (Figure 6; see Methods). We then compared simulated with observed *fog-2* genotype frequency trajectories. The maximum-likelihood (ML) estimates that were most congruent with observations from the sudden populations indicated that outcross-fitness was insufficient to offset the cost of males (ML α =1.41, credible interval (CI) =1.36 to 1.46). Simulations for the gradual populations indicated that during their first 35 generations of evolution, before high salt exposure, upward evolution of α may have occurred, but to a degree still insufficient to offset the cost of males when populations subsequently experienced high salt (ML α =1.76, CI =1.73 to 1.79).

Our modelling of trioecy, with just the outcross-fitness parameter, appears to replicate accurately the experimental transitions to selfing. First, the ML estimate of outcross-fitness in the sudden populations closely matches the observed outcross-fitness value of the lab-adapted androdioecious population (α =1.41, CI =1.36 to 1.46 versus α =1.37 ± 0.06SE, respectively; when the latter is calculated from Figure 2A male frequency changes across two generations [52], while making the same assumptions as above for all other parameters; see Methods). Second, we were also able to recover the observed fitness of the ancestral trioecious population, estimated from the competition assays, when explicitly simulating it with the sudden ML estimate of outcross-fitness (see Additional file 1: Figures S7).

Because our modelling seems accurate, we examined the expected evolution of sex ratios during transitions to selfing. With the ML estimate of the sudden populations, populations would be composed exclusively of hermaphrodites by generation 38 (see Additional file 1: Figure S8). With the ML estimate of the gradual populations, and by the time the experiments were discontinued at generation 68, males would be at about 7% and females at about 0.1%. Therefore, as observed during experimental evolution, for both the sudden and the gradual populations, females were lost from the populations before males and trioecy was not maintained [67-69].

This last analysis suggests that monoecy should be the end reproduction system, as observed in the sudden populations. Particularly in the gradual populations, however, simulations show that potential evolution of outcross-fitness during the period when populations were kept at high salt after generation 35 could have overcome the cost of males. If this was the case, then the evolution of outcross-fitness could have resulted in androdioecy with continued experimental evolution [55,81,85].

### Evolution of male fitness components and the opportunity for reproductive assurance

Possible evolution of outcross-fitness under trioecy raises the interesting possibility that the ancestral dioecious population could have been resistant to the invasion of a hermaphrodite if given enough time for adaptation to high salt before its appearance by mutation (Figure 1A). To partially address this hypothesis, we performed 50 generations of experimental evolution under dioecy in the sudden regime and asked whether the improvement of male fitness components resulted in increased fertility rates, to the extent of reducing the opportunity for reproductive assurance by selfers (see Methods and Additional file 1: Table S1).

**Figure 7:**
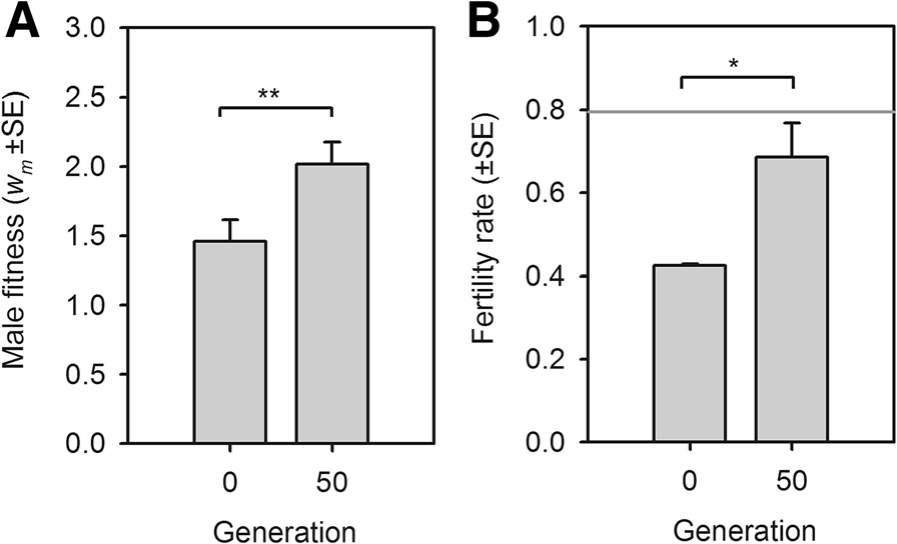
**Evolution of male fitness diminishes the opportunity for reproductive assurance. (A)** Experimental evolution of male fitness in 305 mM NaCl of dioecious populations cultured for 50 generations under the sudden regime. There was a significant increase in male fitness during evolution (LMM: |z| =2.8, *P*-value =0.006, residual d.f. =30). **(B)** Experimental evolution of fertility rates in the sudden dioecious populations. After 50 generations there was an increased proportion of females that were fertile after development from the L1 larval stage (24 ± 2 hours) to the time of reproduction (96 ± 2 hours) in 305 mM NaCl (GLMM: |z| =2.3, *P*-value =0.03, residual d.f. =5). Differences in fertility rates between the evolved G50 dioecious population and the ancestral lab-adapted androdioecious population are not significant (grey line; GLM: |z| =1.6, *P*-value =0.112, residual d.f. =5). For **(A)** and **(B)**, bars and errors show ordinary least-square estimates. *, ** indicates, respectively, *P*-values of <0.05, <0.01. d.f., degrees of freedom; GLMM, generalised linear mixed effects models; LMM, linear mixed effects model.

After 50 generations of dioecious experimental evolution, we estimated ‘male fitness’ in high salt by the relative performance of males when in competition with GFP tester males for the fertilization of *fog-2* tester females and relative progeny survivorship to adulthood (see Methods; GFP males were from the same strain used above in the fertility assays, and *fog-2* females were from an unrelated strain described in [86]). This assay showed that male fitness increased over the course of 50 generations of experimental evolution (Figure 7A). Further assays of dioecious female fertility showed that likely it did not evolve (results not shown).

The improvement in male fitness may be enough to diminish the reproductive assurance that selfers could afford, were they to appear in the dioecious population, if the proportion of females that failed to reproduce in high salt was significantly reduced during sudden experimental evolution (see above). To confirm this, we measured the fertility rates of the dioecious populations at the standard time of passage (96 hours) and compared them to that of the lab-adapted androdioecious population (see Figure 3A). This assay showed that the proportion of fertile females after 50 generations of dioecious evolution in the sudden regime increased from about 40% to about 70% (Figure 7B), a value that was not statistically different from the approximately 80% of hermaphrodites that were fertile in the lab-adapted population. These results indicate that evolution of male dioecious fitness may reduce the opportunity for reproductive assurance. We can, thus, predict that invasions of the ancestral hermaphrodites in the high salt adapted dioecious populations would be more difficult if we were to repeat experimental evolution.

## Conclusions

We have presented comprehensive evidence supporting the hypothesis that individual selection among selfers and outcrossers can drive the transition from dioecy to androdioecy, or to effective monoecy, when there is limited outcrossing in novel environments. Experimental transitions to selfing were due to reproductive assurance, notwithstanding selection of standing genetic diversity, the existence of a cost of males, and the potential for density dependent selection among selfing or outcrossing demes (as we did not model it). Our experiments further suggest that adaptation of dioecious populations, to the environment where outcrossing was once limiting to them, may reduce the opportunity for reproductive assurance that selfers can provide and, as a consequence, the probability of successful transitions to selfing.

Our findings were obtained in highly contrived conditions. Can they help explain transitions to selfing within the *Caenorhabditis* clade? Out of the 38 *Caenorhabditis* species that have been discovered so far, three have probably evolved androdioecy from ancestors that had a dioecious reproduction system [64,65,91]. Extant *Caenorhabditis* species inhabit sparse and ephemeral habitats [92,93], and a metapopulation structure with variable density dynamics is the norm [93,94]. Instead of individual selection, transitions to androdioecy in natural *Caenorhabditis* may have resulted from a correlated response to density dependent selection among demes for dispersal to and/or colonization of novel habitats where outcrossing was not necessarily limited [36,37,39]. The lack of a cost of meiosis in *Caenorhabditis*, since hermaphrodites do not outcross each other, and the existence of a cost of males in outcrossing lineages would then make certain that the first selfing colonizers would outcompete outcrossers reaching the same habitat at a later time, see for example [22] for an empirical example.

Particularly in *C. elegans*, the assumed dispersal life history stage, the dauer, is a stage from which more males than hermaphrodites survive and reach reproductive maturity [58]. Adult males are also more vagile than adult hermaphrodites [95], and several evolution experiments have shown that they can be maintained at intermediate frequencies under a variety of novel environments [55,58-63], some of which with varying density dynamics. These observations suggest that selection among demes for dispersal and/or colonization of novel habitats, not before experienced by the ancestral dioecious populations, would favour males over hermaphrodites and, as a correlated response, outcrossing over selfing. Although it remains to be shown whether, in general, dioecious males likewise survive the dauer stage better and are more vagile than dioecious females, but see [75], selection among demes is not necessary for the transition from dioecy to androdioecy in *Caenorhabditis*. It is sufficient to speculate that in some of the novel environments the ancestral dioecious populations were challenged with restricted outcrossing.

In plants, most of the known transitions from outcrossing to selfing occurred from self-incompatible hermaphrodites that were able to cross-fertilize each other [3,6,26]. In this situation, a mutant self-compatible hermaphrodite that provided reproductive assurance when pollination agents were a limiting factor, and could outcross with other hermaphrodites, should have been favoured over a female-sterile hermaphrodite (pollen-only) [17,27,68]. In these transitions it remains uncertain whether the cost of meiosis, cost of pollen production, selection among selfers and outcrossers or density dependent selection can explain them. Yet, it appears that successful transitions rely on the short-term selective advantage of selfing over outcrossing, thus presumably on individual selection and reproductive assurance [15,19-21]. Since in a few model plants, sex determination mutants can be obtained, and emasculation assays readily done, it would be valuable to perform evolution experiments similar in design to ours to disentangle the relative role of all the relevant processes that can explain transitions to selfing; see for example [96] for experimental evolution under different reproduction systems using *Arabidopsis thaliana*.

In a small number of plants and animals, known transitions from outcrossing to selfing were similar to those of *Caenorhabditis*, from dioecy to androdioecy [68,97,98]. In these rare species, empirical evidence on population density dynamics and on the genetics of sex determination is scarce but individual selection could have been important for transitions to selfing. In the particular case of animals, their populations inhabit apparently homogeneous (aquatic) environments and spawn in large numbers, both circumstances that would be conducive to a lesser role for density dependent selection among demes. Also in common with *Caenorhabditis*, the derived self-compatible hermaphrodites of these androdioecious animals are biased in their allocation of reproductive resources towards the female function, a condition that may reflect the limited mutational options that were available to the dioecious ancestor for the evolution of self-compatibility [97,99,100].

The frequency of transitions to selfing is ultimately a function of mutation rates and of the genetic architecture of sex determination. In the dioecious *C. remanei*, at least two mutational steps are required to turn a wild-type female into a self-compatible and fertile protandrous hermaphrodite: one to repress oogenesis and initiate spermatogenesis, and another one to stop spermatogenesis, resume oogenesis and allow fertilization [29,30]. In addition, partially to complete recessive alleles at several loci may have determined autonomous selfing in the ancestral dioecious *Caenorhabditis* [31], a situation that likely would have reduced the probability of the establishment of hermaphrodites, especially if populations during transitions were small or highly structured [101]. In spite of the genetic architecture of selfing, however, as shown here, once these mutants escape genetic drift and became established in the resident population [70], their fate due to selection would be contingent upon whether the ancestral dioecious population was already adapted to the environment where outcrossing was once limiting to them. The opportunity for reproductive assurance may, therefore, be circumscribed to very short evolutionary periods, which, together with limited and probably complex mutational options determining self-compatibility, can help explain why transitions from dioecy to androdioecy are the rarest of transitions to selfing in nature.

## Methods

### Androdioecious populations

All populations reported here were derived from a population with standing genetic diversity previously evolved for 140 generations under our standardised laboratory environment [55,70]. A schematic of the lab environment is shown in Figure 1B. This lab-adapted population is designated EEVA6140, where EEV stands for the *Wormbase* lab acronym, A for androdioecy, and 6140 for 140 generations of experimental evolution of replicate population #6. We drop EEV henceforth.

In order to maintain similar levels of genetic diversity among the ancestor populations used here, 500 A6140 hermaphrodites were individually mated with an excess of A6140 males. All F1 lineages were then expanded in numbers for two generations. A total of 444 of these were recovered, mixed in equal proportions and cryogenically frozen at -80°C at high densities [102]. This androdioecious population is named A00.

### Trioecious and dioecious populations

The *fog-2(q71)* allele located in chrV:25 cM was introgressed in A6140 in two stages, the first with the goal of obtaining homozygous lineages for this allele in a A6140 genetic background, and the second with the goal of introducing A6140 genetic diversity.

In the first stage of the introgression, 50 *fog-2(q71)* homozygous females were recovered from a 100 generation lab-adapted male-female population (D2100; described in [55,71], and individually crossed to an excess of A6140 males. F1 hermaphrodites were selfed to generate F2s, while the F1 male progeny were kept at 4°C in order to be used in subsequent crosses. F2 progeny were collected two days later as immature L4 staged larvae and scored for *fog-2(q71)* homozygosity by the accumulation of unfertilised oocytes during the next two days [80]. From each of the fifty F1 lineages, eight F2 females were outcrossed with an excess of F1 males from a different lineage, and their F3 female progeny mated with one sibling male to generate the F4s. F3 parents were PCR genotyped to confirm homozygosity at the *fog-2* locus (see below). Only a small region surrounding the *fog-2* locus is expected to contain the N2 wild isolate genetic background from where the *fog-2(q71)* allele is ultimately derived [80] (results not shown). This is because the *fog-2* locus is located in the telomere and the origin of the allele for the introgressions is from a lab-adapted population with standing genetic diversity.

In the second stage of the introgression, 12 females from each of 50 F1 lineages were outcrossed with an excess of A6140 males. A total of 600 F1 hermaphrodites were selfed and F2 progeny scored for *fog-2(q71)* homozygosity. A total of 600 F2 females were next mated with males randomly coming from the 50 F1 lineages. The F3s were PCR genotyped and the wild type allele was estimated to be at 5.2 × 10^-2^. F3 females were mated with sibling males to generate the F4s. Since we did not control for the homozygosity of *fog-2* in males, the wild type allele in the F4s is expected to segregate at 2.6 × 10^-2^. A total of 444 F4 lineages were recovered, separately grown to high densities and mixed in equal proportions to constitute the ancestral trioecious population, named T00. Samples were frozen at -80°C.

To derive the ancestor dioecious population (D00), 350 adult T00 females were individually mated with single T00 males, after scoring for the accumulation of unfertilised eggs in them. Twenty-four hours later, males were genotyped at the *fog-2* locus and 318 lineages were recovered where both parents were *fog-2(q71/q71).* These lineages were grown to high densities, mixed at equal proportions and samples frozen at -80°C.

### Monoecious population

To derive the ancestor monoecious population (M00), the *xol-1(tm3055)* allele located in chrX:-0.45 cM was introgressed in A6140 in two stages.

In the first stage, parental hermaphrodites from strain FX03055 [103] were mated with an excess of A6140 males. F1 progeny were separately selfed, and with PCR genotyping, F2 hermaphrodites were confirmed to be *xol-1(tm3055)* homozygous after laying of the F3 embryos (see below). Lineages were then kept for another generation. This four-generation cycle was repeated another two times, with the parental hermaphrodites coming from a homozygous *xol-1(tm3055)* F3 lineage obtained in the first cycle. Ten crosses were done in the first cycle and twenty in the second and third cycles. At the F1 generation, 70, 286 and 378 hermaphroditic lineages were obtained in the first, second, and third cycle, respectively. At the F2 generation, 3,800, 1,228 and 1,219 were obtained in the first, second, and third cycle, respectively. For the F3 generation we obtained 752, 350 and 1,144 lineages in the first, second, and third cycle, respectively. At the end of each cycle, F3 individuals were genotyped at 57 bi-allelic SNPs spanning the X chromosome (see below and Additional file 3 for SNP information). Two lineages containing less than 0.5 Mbp of the N2 wild isolate genetic background surrounding the *xol-1* locus were recovered (results not shown).

In the second stage of the introgression, 300 hermaphrodites from each of the two lineages recovered after the first stage were individually mated with an excess of A6140 males. Their F1 progeny were selfed to generate 4,800 F2 hermaphrodites, each then being selfed in order to obtain the F3s and, subsequently, the F4s. Each of the 4,800 F2s was collected after reproduction for PCR genotyping. A total of 444 F2 homozygous *xol-1(tm3055)* lineages were recovered. They were grown to high densities, mixed in equal proportions and samples frozen at -80°C.

### Green fluorescent protein testers

As previously described [70], a fully penetrant and genome-integrated GFP marker with a *myo-3* promoter was introgressed in the A6140 population to obtain a tester population with standing genetic diversity (A6140GFP). Heterozygous and homozygous genotypes similarly express GFP in all muscle cells. A6140GFP is here used in the (population-wide) fitness assays (see below; Figure 4, Additional file 1: Figures S3-S5).

As previously described [70], A6140GFP hermaphrodites were selfed for 12 generations, in order to obtain highly inbred strains. One of these strains (A6140GFPL1) is here used in the hermaphrodite/female fertility and male fitness assays (see below; Figures 2 and 6).

### Experimental evolution under different salt regimes

Frozen samples with >10^4^ individuals of the A00, T00, D00 and M00 populations were thawed and after one passage, 10^4^ individuals were seeded for each of *rR*1-# replicate populations; where *r* designates the salt regime (*S*udden, *G*radual or *C*ontrol), *R* the reproduction system (*A*ndrodioecy, *T*rioecy, *D*ioecy, *M*onoecy), and *#* the replicate population number. Dioecious populations were cultured only in the sudden regime. A total of 43 populations underwent experimental evolution (see Additional file 1: Table S1 for full designations).

Following our standard laboratory environment (Figure 1B) [55], populations were kept in ten 9 cm Petri plates with 28 mL of solid NGM-lite media (Europe Bioproducts, Cambridge England) covered by an overnight grown lawn of HT115 *Escherichia coli*. Bacteria provided *ad libitum* food for worm development from the L1 larval stage until adult reproduction. NGM-lite media contains NaCl at 25 mM (0.14% w/v). At 24 ± 2 hours of the life cycle, each population was seeded with 1,000 first larval staged (L1) individuals in each of the 10 Petri plates. After development to maturity for 72 ± 2 hours at constant 20°C and 80% relative humidity (RH), all 10 plates were mixed and harvested worms exposed to 1 M KOH: 5% NaOCl ‘bleach’ solution for 5 minutes ±15 seconds, to which only embryos survive [102]. After repeated washes with the M9 isotonic solution, embryos were maintained in a shaker in 3 to 5 mL M9 in 15 mL conical tubes, at 20°C and 120 rpm. After 24 ± 2 hours, adult debris was removed after centrifugation at 200 rpm and the density of live L1s estimated under a dissection scope. The appropriate M9 volume with live L1s was then placed in fresh NGM-lite plates to complete one life cycle.

The control regime was the same in which the lab-adapted population had been cultured for 140 generations. In particular, NGM-lite media in the plates were not supplemented with NaCl. Replicates were cultured for 68 generations. The sudden regime was characterised by the same standard conditions, except that the NGM-lite plates were supplemented to 305 mM NaCl from the start of evolution. NaCl was dissolved in ddH_2_O and the appropriate volume added to NGM-lite before autoclaving (1.78% w/v, minimum 99% purity, Roth P029.3). ST, SM and SA populations were cultured for 38 generations, SD populations were cultured for 50 generations. For the gradual regime NGM-lite plates were supplemented with increasing concentrations of NaCl from 33 mM at generation 1 to 305 mM NaCl at generation 35 and onwards. Replicates were cultured for 68 generations. With the exception of SD1-4, all other populations concurrently underwent experimental evolution. Samples from each population were periodically stored at -80°C at high densities (>10^3^) for posterior characterisation (see Additional file 1: Table S1).

### Male frequency assay in A6140

After thawing frozen samples of A6140 in 25 mM NaCl NGM-lite plates, worms were passaged twice as during experimental evolution. The parental P generation was then seeded into either 25 mM plates or 305 mM NaCl plates, in triplicate, at the L1 larval stage. At 96 ± 4 hours of the life cycle, 300 adult individuals were sexed under a dissection scope at 15× magnification, following random trajectories and covering the whole surface of the plate. Samples from each plate were separately cultured to generate the F1 generation, which was similarly maintained at either 25 mM NaCl or 305 mM NaCl and sexed when individuals reached adulthood. The assay was repeated eight times (blocks). As the data is proportional, for analysis we did generalised linear mixed effects models (GLMM) with binomial (logit-link) errors to estimate fixed differences between the P and F1 generation at each environment or to estimate fixed differences between environments at the F1 generation [104]. Only the later analysis is reported. Block was modelled as the random independent variable. Significance of effects was assessed with z-ratio tests. The function *glmer* in the package *lme4* within R was used for computations [105,106]. In one additional assay, we further detailed the effects of NaCl on male proportions (see Additional file 1: Figure S1). These assays were done over two generations in triplicate in NGM-lite plates containing 150 mM, 250 mM, 275 mM or 305 mM NaCl.

### Male frequency assay in experimentally evolved populations

Ancestral (A00, T00) and evolved (rA1-3, GT1-3, ST1/3/5) populations were measured for male proportions once experimental evolution was completed (see Additional file 1: Table S1). The assay was done in 12 blocks, to include samples for generations 5, 15, 25 and 35 from the sudden regime, generations 35, 50, 56 and 68 from the gradual regime, and finally, generations 15, 35 and 68 from the control regime. Frozen samples of >10^5^ individuals in each population were thawed and passaged for two generations in 25 mM NaCl NGM-lite plates. In the second generation, at 96 ± 4 hours of the life cycle, 300 individuals were sexed per each of three plates per population sample. We did not statistically model male frequency experimental evolution.

### *fog-2* genotype frequency assay

Frozen samples were thawed and passaged once in 25 mM NaCl NGM-lite plates. In the second generation, 48 L3 or L4 larval staged individuals per population and experimental regime were PCR genotyped at the *fog-2* locus (see below). Samples from generations 5, 15, 25 and 35 were chosen for ST1/3/5 populations, generations 35, 50, 56 and 68 for GT1-3 populations and generation 23 for CT1-3 populations (see Additional file 1: Table S1). In each PCR, positive and negative control samples were included with gDNA of individuals with known genotypes that were constructed prior to the assays. After quality control of the data, we had an average of 42.7 ± 4SD observations per population and at each time point. Assuming binomial sampling distributions, we can thus detect wild type allele frequencies above 2% with a statistical power of 0.8. Note that since we did not detect the *fog-2(wt)* allele in CT1-3 at generation 23, it is impossible for transitions to occur by genetic drift in subsequent experimental evolution [70].

The ‘transition fitness’ of the *fog-2(wt)* allele (*s*), the invading allele in the trioecious populations, can be calculated as its expected deterministic frequency change over the resident *fog-2(q71)* allele [70,82]. Note that transition fitness is different from ‘invasive fitness’ since the invader allele is at relatively high frequencies during most of experimental evolution, compare with [82,83]. We employed ANOVA to estimate *s* as the fixed (continuous) effects of generation on the natural log ratio changes of *fog-2(wt)/ fog-2(q71)* allele frequencies, assuming random mating and selfing and no sex ratio segregation distortion (see Additional file 1: Table S2). When we failed to detect the *fog-2(wt)* allele (for example, generation 5 of the sudden treatment), we considered it at a frequency of 1/97, while when all samples were positive, we considered it at a frequency of 96/97. This allowed us to calculate the log ratios for an initial sample size of 48 individuals while being conservative. The *lm* function in the *stats* package of R was used for calculations [105].

### Competitive fitness assay

We assayed fitness of all evolved populations at generation 35 (rT1-#, rM1-#, and rA1-#), and the three ancestral populations (T00, M00, A00; Additional file 1: Table S1). For this, we performed head-to-head competitions between experimental populations and the A6140GFP tester population. For each competition, more than 10^3^ individuals from each competitor were thawed from -80°C in parallel and expanded in numbers for two generations in 25 mM NaCl NGM-lite plates. On the third generation, L1-staged GFP individuals were mixed with wild type experimental individuals at expected relative frequencies of 50%; see [70] for a density calibration curve of this protocol. Worms then developed, matured and reproduced in NGM-lite plates supplemented to 305 mM NaCl. For T00, M00 and A00 we also followed the same protocol and assayed them at 25 mM NaCl (results presented in Additional file 1: Figure S5). GFP scoring was done in the following generation, after bleaching and 24 ± 2 hours of maintenance in M9, by photographing 5 μl of M9 containing live L1 larvae on a glass slide, at a resolution of 1.5 pixel/μm under a fluorescent dissection scope equipped with a digital camera.

The competition assays were done in 12 blocks, each having a different thawing day. In each block, the wild type experimental populations, GFP tester and the A6140 populations were thawed in parallel, such that a total of 12 independent competitions between the GFP tester and the A6140 population were also obtained (see Additional file 1: Figure S3). Each competition was replicated between four and six times, with 3 × 10^3^ individuals (three plates) being employed per replicate. All replicates started from the same mix of GFP and wild type experimental individuals. Setup and scoring was randomised across treatments and experimenters within block. The mean number of GFP/wild type L1s scored per population sample after one generation of competition was of 330/902 with the 2.5% and 97.5% quantiles being of 92/243 and 1,031/2,618 individuals, respectively. Frequency estimates of wild type and GFP alleles were also obtained for the mixes at setup (see Additional file 1: Figure S4).

Relative changes in the wild type allele frequencies over the GFP allele in each competition, measured at the L1 stage and in two consecutive generations, are expected to reflect variation in all fitness components: L1 to adult developmental time and survivorship, mating, fertilization, fecundity and embryo to L1 hatching success. Without loss of generality, a haploid fitness coefficient can be estimated as the change in ratio in one generation of the wild type non-GFP allele over the tester GFP allele [70,83,85]: w = ln (p_wt.t1_/p_GFP.t1_) – ln (p_wt.t0_/p_GFP.t0_); where *p*_*wt*_ and *p*_*GFP*_ are the wild type and GFP allele frequencies, respectively, at setup *t0*, and after the competition, *t1*. Note that this fitness coefficient is defined in a similar way as the transition fitness coefficient (see above) [82,83].

Since the scoring procedure employed in the assay was based on the presence/absence of GFP expression, we were unable to score for progeny heterozygosity. To correct for this, an algorithm was written in R taking into consideration the male frequencies from each of the two competing populations (which were separately measured during the assay following the above described protocol) and the starting and final GFP counts in order to retrieve the expected number of alleles after the competition. This algorithm assumed random mating and selfing, and no sex segregation distortion [52] (see Additional file 1: Table S2). The GFP allele does not have a fitness cost over the wild type allele of the A6140 population when in high salt (see Additional file 1: Figure S3).

Due to the high heterogeneity among blocks and because at a given block not all experimental populations were assayed (see Additional file 1: Figure S3), the fitness estimates of the experimental populations obtained at a given block were transformed by subtracting the average *w* of the A6140 population for the corresponding block (*w*_*t*_). Quality control of *w*_*t*_ data involved removing from analysis all estimates from the ST2 population because there was a significant deviation from the expected 50:50 L1 mix of wild type: GFP at assay set up that likely determined the observed negative fitness (see Additional file 1: Figure S4; see [70] for a demonstration of a change in the sign of fitness coefficients because of frequency dependence, using similarly designed assays).

Analysis of *w*_*t*_ involved two steps, the first with the goal of obtaining the ordinary least-square estimates of the three ancestral populations (T00, A00, M00) and the second with the goal of determining the extent of fitness responses after 35 generations of evolution in all derived populations (G35; rT1-#, rM1-# and rA1-#; Additional file 1: Table S1), which we define here as ‘adaptation’ or ‘adaptive rates’ to the high salt environment. For the first step, *w*_*t*_ was modelled as a function of the fixed reproduction system with ANOVA (see Additional file 1: Figure S5). We employed the function *lm* within the *stats* package in R for calculations. The least-square estimates obtained per ancestral population were then subtracted from the transformed data of the corresponding G35 experimental populations for subsequent analysis (*Δw*_*t*_; Figure 4). For each reproduction system, *Δw*_*t*_ data were modelled as a function of the fixed salt regime (sudden, gradual or control) and random replicate population, with linear mixed effects models (LMM) using maximum likelihood methods [104]. We did not model the ratio of wild type to GFP alleles at setup since preliminary models did not find it to be significant (not shown). Significance of fitness responses at each regime were assessed with z-ratio tests. We employed the *lmer* function within the *lme4* package in R for calculations [106]. *Post hoc* comparisons among salt regimes within each reproduction system were done with Tukey tests and assuming Student t distributions where the degrees of freedom were asymptotically determined, using the *lsmeans* package in R [107].

### Selfed versus outcrossed hermaphrodite fertility assay

We measured the fertility of A6140 hermaphrodites when selfing and when outcrossing occurs in high or low salt (Figure 2). Stocks were thawed and in parallel passaged once in 25 mM NaCl NGM-lite plates. In the second generation, 50 L4 larval stage (reproductively immature) hermaphrodites were transferred to 9 cm 25 mM NaCl plates and mated with an excess of males coming from the A6140GFP inbred tester strain. GFP outcrossed progeny grew on these plates and as L4s they were individually transferred to 6 cm NGM-lite plates, with either 25 mM or 305 mM NaCl, and placed together with two A6140GFPL1 tester males or allowed to self without the presence of males. Parents were removed from the plates 24 ± 2 hours later, and three to four days later the number of adult viable progeny and GFP status was scored. The assay was done in two blocks. Setup and scoring was randomised across treatments and experimenters.

In order to compare selfed and outcrossed treatments, we eliminated all observations where less than five progeny were scored (eliminated 66 observations), and when less than 10% of male progeny in the outcrossed treatment were observed (eliminated 14 observations). We tested for the fixed effects of breeding mode and salt environment on fertility with GLMMs by taking block as the random independent variable. We considered that the model error followed a Poisson distribution by using the log-link function, since the data were scored as counts. Least-square estimates were obtained by maximum likelihood. z-ratio tests were done to test for significance of fixed effects and Tukey *post hoc* z-tests were done to test for differences in breeding mode in each salt environment. For calculations we employed the *glmer* function within the *lme4* package in R [104,106]. For plotting, ordinary least-square estimates are presented.

### Hermaphrodite and female fertility assay

A6140 hermaphrodites and D00 females were measured for fertility when outcrossed to D00 males after development since the L1 larval stage in high salt conditions (Figure 3). A6140 and D00 stocks were thawed and passaged twice in parallel in 25 mM NaCl NGM-lite plates. On the third generation, L1 individuals were seeded in two 305 mM NaCl NGM-lite plates. After 72 ± 2 hours of development, A6140 hermaphrodites and D00 females were each individually placed with two D00 males in 6 cm 305 mM NaCl NGM-lite mating plates with 5 uL of *E. coli*. All adults were removed 24 ± 2 hours later and the plates left to incubate in standard conditions. After four days all progeny was scored for number.

Preliminary analysis showed that data including zero fertility followed a negative binomial distribution (for A6140, central tendency ‘mean’ ± CI =1.5 ± 0.3; for D00, mean =3.74 ± 0.83; both similar dispersion parameters; calculated with the *fitdistr* function in the *MASS* package in R [104]). For this reason, we decided to eliminate all zero fertility observations (for A6140, we eliminated 24 observations; for D00, 12) and then tested for differences among populations with a generalised linear model (GLM) employing a Poisson distributed error. A z-ratio test was done to test for significance of effects. For plotting, ordinary least-square estimates are presented.

### Developmental time to maturity and population fertility rate assay

By measuring the population fertility rate at several time points during the standard life cycle (Figure 1B), we were able to estimate developmental time to maturity in A6140 under conditions of low and high salt (Figure 2). For D00 and SD1-4, we only estimated fertility rates at one time point (Figures 3 and 7). All populations were concurrently assayed.

A6140, D00 and SD1-4 stocks were thawed and passaged twice in parallel in 25 mM NaCl NGM-lite plates. On the third generation, 1,000 L1s were seeded either in three 25 mM NaCl NGM-lite plates or in three 305 mM NaCl NGM-lite plates. For A6140, we hand-picked 24 hermaphrodites after 48 ± 2 hours or 64 ± 2 hours from the L1 seed, from each of the three plates in both salt conditions, and placed them in 12-well cell culture plates with 3 mL 25 mM or 305 mM NaCl NGM-lite and with 5 uL of *E. coli*. Two hours later, wells were scored for the presence of fertilised embryos (identified by their non-transparent appearance). For D00 and SD1-4, similar numbers of individuals were picked at 64 ± 2 hours after L1 seed and growth in 305 mM NaCl NGM-lite plates. Setup and scoring was randomised across treatments.

For analysis of A6140 data, we employed GLM on the count data of fertile to infertile individuals per assay plate and tested for the fixed effects of time of life cycle and NaCl condition (Figure 2). A6140 and D00 data were similarly compared at 305 mM NaCl with GLM (Figure 3). Experimental evolution responses in 305 mM NaCl in SD1-4 were compared to the values of D00 with a GLMM for the fixed effects of generation and the random effects of replicate population (Figure 7). For all models binomial errors were considered by using the logit link function (as the data is proportional data) and z-ratio tests were done to test for significance of effects. We employed *glm* or the *glmer* functions within the *stats* or the *lme4* packages in R, respectively, for calculations. For plotting, ordinary least-square estimates are presented.

### Male fitness assay

Generation 50 SD1-4 and ancestral D00 stocks were thawed and grown in parallel under standard 25 mM NaCl conditions for two generations. At 72 ± 2 hours of the third generation, nine experimental males were transferred to 6 cm 305 mM NaCl NGM-lite plates spotted with 5 uL *E. coli*, together with nine A6140GFPL1 tester males and twenty-two day 4 adult *fog-2(q71/q71)* females (from the inbred line D0L27, described in [70]). An average of 16.9 ± 4.2SD females per mating plate were transferred to a new plate 24 ± 2 hours later and killed with 30 μL of the bleach solution. These plates were incubated during the next three to five days at 20°C and 80% RH, and the viable adult progeny scored for GFP expression at 30× magnification under a stereoscope. Five replicate mating plates were done per population and all populations assayed over four blocks. Setup and scoring was randomised across treatments.

Quality control of the adult progeny count involved discarding mating plates where less than six females were transferred and also those where less than twenty progeny were scored. Similarly to transition fitness and (population-wide) fitness (see above) per mating plate, a male haploid fitness coefficient was defined as: w_m_ = ln(p_t1_/(1-p_t1_))- ln(p_t0_/(1-p_t0_)); with *p*_*t0*_ being the fixed 0.5 wild type allele frequencies at the set up generation and *p*_*t1*_ the observed wild type allele frequencies after the competition [85]. We assumed random mating and no sex ratio segregation distortion. For analysis we conducted LMM by taking the number of females from which progeny was scored as a continuous covariate, generation as a fixed independent variable and block as the random independent variable (Figure 7). z-ratio tests were done to determine the significance of evolutionary responses. The *lmer* function within the *lme4* package in R was employed for calculations.

### Simulations of *fog-2* genotype frequency dynamics

We numerically simulated the expected change in the frequency of the f*og-2(wt)* allele during trioecious experimental evolution in the sudden and gradual regimes as a function of the relative outcross-fitness parameter (*α*). Despite following the previous *C. elegans* modelling of androdioecy [52,90], we updated it to the expected reproduction under trioecy, as detailed in Additional file 1: Table S2.

Simulations started with N = 10^4^ individuals, where the proportion of the different sexes and breeding modes were given by the observed allele frequencies at the *fog-2* locus in the rT1/3/5 populations (see Additional file 1: Table S1). *fog-2* genotype frequencies in males were assumed to be the same as those of hermaphrodites/females. *αm* matings occurred, with *α* being outcross-fitness and *m* the observed male frequency, indiscriminately between males and hermaphrodites or between males and females. Mating pairs were randomly selected by collecting either *αmN* females and hermaphrodites or the total number of females and hermaphrodites (the number that was lower), together with the same number of males. Males were sampled with replacement. During mating, we followed genotype identity for subsequent reference. If *αmN* was less than the total number of hermaphrodites and females, then unmated individuals were allowed to constitute pseudo mating pairs with probability 1 if they were hermaphrodites and 0 otherwise. This means that the β parameter of [52], which defined the success rate of the non-outcrossed gamete partition, was set to one under selfing. Accounting for these pseudo mating pairs was necessary to ensure Mendelian segregation at *fog-2* under selfing. At the next stage, all of the mating pairs and pseudo mating pairs were sampled with replacement until a new population with N individuals was obtained. The *fog-2* genotypes were reconstituted by random sampling of alleles from each of the parental genotypes. Half of the individuals that resulted from outcrossing events were defined as males. The remaining individuals, irrespective of being generated by outcrossing or selfing, were defined as hermaphrodites or females depending on their *fog-2* genotype.

Estimates of the outcross-fitness parameter (*α*) congruent with the observed *fog-2* genotype frequency dynamics were obtained under a likelihood framework, whereby *fog-2(wt)* allele counts over time provided the probability of the α-values. Simulations were done independently for each time period sampled during the evolution. For the sudden populations there were three periods: from generation 5 (G5) to G15, from G15 to G25, and from G25 to G35. For the gradual populations there were three periods: from G35 to G50, G50 to G56, and G56 to G68. Twenty replicate simulations were performed per starting conditions and for each combination of α between 0 and 2 in a grid of 51 points. The probability of the observed *fog-2(wt)* allele count data given the simulated mean frequencies was obtained from a binomial distribution. The natural logarithms of these probabilities were summed across periods, and the compound value with the higher likelihood was taken as the ML estimate. CIs were defined by -2lnLk around the ML (Figure 6).

### Simulated fitness of T00

With the same simulation algorithm, fitness (*w*_*t*_) of T00 was estimated for one life cycle of competition with the GFP tester population, assuming fixed setup of 50 wild type to 50 GFP alleles (see Additional file 1: Figure S8). For several α values we obtained the sex ratios after one generation of competition and estimated allele frequencies assuming random mating and selfing and no sex ratio segregation distortion. For the results presented in Additional file 1: Figure S7, an inbreeding depression parameter (δ) was introduced as a sampling weight before reproduction of GFP hermaphrodites relative to the wild type trioecious females, according to the *C. elegans* androdioecious model (see main text and [52]).

### Estimates of outcross-fitness in A6140

Outcross-fitness (*α*) was calculated for the lab-adapted A6140 population from the male frequency changes observed after two generations of culture in 305 mM NaCl (from Figure 2A). We followed the ‘male maintenance function’ of [52]: m_t1_ = αm _t0_/(2αm _t0_ + β(1-αm_t0_)), with m_t0_ being the male frequency after one generation in high salt (P) and m_t1_ the male frequency after two generations (F1).

### DNA extraction

Genomic DNA was prepared from single immature L3 or L4 larval staged individuals using the prepGEM Insect kit (ZyGEM), following standard protocols [108].

### PCR genotyping

For the introgression of *xol-1(tm3055)* in A6140 (see above), we used PCR genotyping with the forward primers XOL2L: 5′-GGGATTGATAGGAGCGAAA-3′, XOL4L: 5′-ATGATTGATGATTTACCGAAGC-3′ and the reverse primer XOL2R: 5′-GGGATTGAGCACCAAACTT-3′. *xol-1 (wt/wt)* individuals yielded a single 471 bp band, *xol-1 (tm3055/tm3055)* one 375 bp band, and the heterozygotes both. PCR amplifications were carried out under the following conditions: 50 to 150 ng of genomic DNA were used in a 15 μl reaction that contained 3 μl Green GoTaq© Flexi Buffer 10× (Promega, Madison USA), 1 mM MgCl2, 0.3 mM of primer XOL2L, 0.5 mM of primer XOL4L, 0.5 mM of primer XOL2R, 0.2 mM of each dNTP, and 0.625 units of GOTaq DNA polymerase (Promega, Madison USA). Typically, reactions went through 35 cycles, after an initial denaturation of 3 minutes at 95°C under the following conditions: 30 seconds at 95°C, 30 seconds at 59°C, 45 seconds at 72°C, followed by an extension step at 72°C for 3 minutes.

To score *fog-2* genotype frequency dynamics (see above), we used PCR genotyping with the forward primers F1RFLP: 5′-CTGTCCAGATACGCCTCTCGTCT-3′, FogR4short: 5′-CTGATTGAGCAATATGTCGAATT-3′ and the reverse primer FogR3: 5′-ACGCCTGTGTGAAATTGGGCAAAAGATTAGACTGATTGAGCAATATCGATAATC-3′. *fog-2(wt/wt)* individuals yielded one 295 bp DNA band visualised in agarose gels, while *fog-2(q71/ q71)* gave one 264 bp band. Heterozygotes exhibited a double band pattern. PCR amplifications were carried out under the following conditions: 50 to 150 ng of genomic DNA were used in a 15 μl reaction which contained 3 μl Green GoTaq© Flexi Buffer 10×, 1.5 mM MgCl2, 0.5 mM of primer F1RFLP, 0.75 mM of primer FogR4short, 0.25 mM of primer FogR3, 0.2 mM of each dNTP, and 0.625 units of GOTaq DNA polymerase. After 3 minutes at 95°C, 35 cycles were done each with 30 seconds at 95°C, 30 seconds at 58°C, 30 seconds at 72°C. A final step was done at 72°C for 3 minutes.

*Wormbase.org* genome release WS220 was used to design the PCR oligos.

### SNP genotyping

To determine standing levels of genetic diversity, SNPs were genotyped in 39 populations at generation 22 of experimental evolution and in the ancestral lab-adapted population (see Additional file 1: Table S1). Sixty-nine bi-allelic SNPs along chromosome IV were chosen and genotyped with the iPlex SequenomTM MALDITOF platform following previously described protocols [55,71]. WS200 was used for the oligonucleotide design employed for PCR amplification followed by allele-specific extension. Oligonucleotide information is available from the authors upon request.

For data analysis, we excluded SNPs with more than 80% missing data across all samples followed by removal of individuals with more than 60% of missing data. In a final step, SNPs with more than 50% of missing data were also removed. The resulting data include genotypes for 58 SNPs. SNP information and sample sizes are presented in Additional file 2.

For the introgression of the *xol-1(tm3055)* allele in A6140 (see above), 57 bi-allelic SNPs along chromosome X were also genotyped with the iPlex SequenomTM MALDITOF platform (see Additional file 3).

### SNP diversity analysis

For each population sample, we calculated homozygosity as one minus the average observed number of heterozygous genotypes across SNPs (*1-Ho*). We also calculated pairwise SNP linkage disequilibrium (*r*^*2*^) as the composite genotype disequilibria, assuming that the genotype probabilities are the products of the gametic probabilities [109]. SNPs with minor allele frequencies of <0.05 were removed prior to analysis to prevent low sample size bias [110]. Because of this correction, we failed to calculate *r*^*2*^ for SM4 and SM7 (see Additional file 1: Table S3).

To test the dependency of fitness responses at generation 35 (*Δw*_*t*_) on homozygosity (*1-Ho*) or linkage disequilibrium (*r*^*2*^) at generation 22, we did ANCOVAs (Table 1). *1-Ho* or *r*^*2*^ were taken as the continuous independent covariate and reproduction system (trioecy, monoecy, androdioecy) as the categorical fixed independent variable. The ST2 genotype data were not included in the analysis. Modelling reproduction system and salt regime as categorical fixed independent variables gave similar results (results not shown). Expanding the scale of *1-Ho* or *r*^*2*^, for example by angular transformation, also produces similar results (results not shown). The *lm* function within the *stats* package in R was used for calculations.

### Availability of supporting data

The data supporting the results of this article are available in the *Dryad.org* repository, doi:10.5061/dryad.4013. The data are composed of the male and *fog-2* genotype frequency data, fitness data, male fitness, fertility data and SNP genotype data. The R scripts used for numerical modelling can be obtained from the authors upon request.

## Additional files

Additional file 1:
**Supplementary information and results.** PDF file containing eight figures and three tables. **Figure S1.** Male frequencies in the lab-adapted population. **Figure S2.** Embryo to adulthood hermaphrodite survivorship. **Figure S3.** Fitness of wild type over GFP alleles. **Figure S4.** Quality control for fitness data. **Figure S5.** Fitness of ancestral populations. **Figure S6.** Evolution of male frequencies under androdioecy. **Figure S7.** Expected fitness of the ancestral trioecious population. **Figure S8.** Expected sex ratios during transitions to selfing. **Table S1.** ID of replicate populations and assays. **Table S2.** Expected reproduction table under trioecy. **Table S3.** SNP sample sizes employed to calculate *r* ^*2*^. Additional file 2:
**SNP table for chromosome IV.** Text file containing SNP information and the sample sizes for each of the populations genotyped at generation 22 of experimental evolution. Additional file 3:
**SNP table for chromosome X.** Text file containing information about the SNPs genotyped for the construction of the monoecious population M00.
