# Supplementary material for: Reproductive assurance drives transitions to self-fertilization in experimental Caenorhabditis elegans
Source: BMC Biol. 2014 Nov 5;12:93. doi: 10.1186/s12915-014-0093-1 (PMC4234830; doi:10.1186/s12915-014-0093-1)
Supplement: Additional file 1: — Supplementary information and results. PDF file containing eight figures and three tables. Figure S1. Male frequencies in the lab-adapted population. Figure S2. Embryo to adulthood hermaphrodite survivorship. Figure S3. Fitness of wild type over GFP alleles. Figure S4. Quality control for fitness data. Figure S5. Fitness of ancestral populations. Figure S6. Evolution of male frequencies under androdioecy. Figure S7. Expected fitness of the ancestral trioecious population. Figure S8. Expected sex ratios during transitions to selfing. Table S1. ID of replicate populations and assays. Table S2. Expected reproduction table under trioecy. Table S3. SNP sample sizes employed to calculate r 2. [file 12915_2014_93_MOESM1_ESM.pdf]

Additional file 1 – Supplementary information and results

Reproductive assurance drives transitions to self-fertilization in experimental Caenorhabditis elegans

Ioannis Theologidis, Ivo M. Chelo, Christine Goy, Henrique Teotónio

Correspondence to: teotonio@biologie.ens.fr

Contents:

Page 2 - Figure S1. Male frequencies in the lab-adapted population.

Page 3 - Figure S2. Embryo to adulthood hermaphrodite viability.

Page 4 - Figure S3. Fitness of wild-type over GFP alleles.

Page 5 - Figure S4. Quality control for fitness data.

Page 6 - Figure S5. Fitness of ancestral populations.

Page 7 - Figure S6. Evolution of male frequencies under androdioecy.

Page 8 - Figure S7. Expected sex ratios during transitions to selfing.

Page 9 - Figure S8. Expected fitness of the ancestral trioecious population.

Page 10 - Table S1. ID of replicate populations and assays.

Page 11 - Table S2. Expected reproduction table under trioecy.

Page 12 - Table S3. SNP sample sizes employed to calculate r^2.

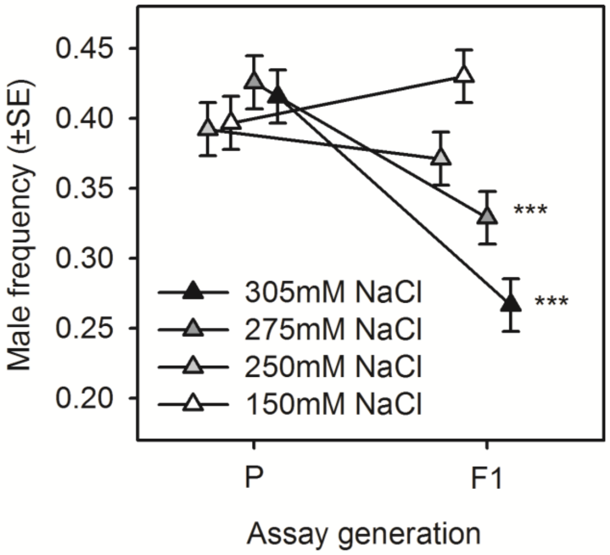

Figure S1. Male frequencies in the lab-adapted population at several NaCl concentrations.

In the lab-adapted androdioecious population, growth from the L1 larval stage (24h±2h) to the time of reproduction (96h±2h) in varying NaCl concentrations did not affect male frequencies (parental generation, P; analysis not shown). With NaCl concentrations above 275mM F1 male frequencies were reduced. Generalized linear models were employed to analyse the male: hermaphrodite count data, separately per NaCl condition. Differences between P and F1 were tested with z-ratio tests while assuming binomial error distributions (all models with residual d.f. = 4). *** indicates p-values<0.001. Similar results are obtained when testing for differences at F1 among NaCl concentrations. For plotting we show the ordinary least-square mean and error estimates of an ANOVA model taking assay generation and NaCl conditions as fixed factors.

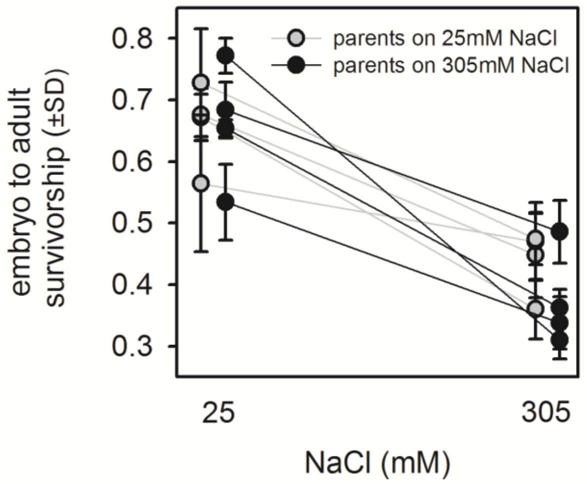

Figure S2. Embryo to adulthood hermaphrodite viability.

Embryo to adulthood viabilities were tested in inbred lines (circles and connecting lines) derived by 12 generations of selfing of hermaphrodites from the lab-adapted androdioecious population (line designations: A6140L126, A6140L188, A6140L142, A6140L244). For the assay, frozen stocks were thawed and passaged for two generations under the standard 25mM NaCl conditions (see Methods). At the third generation after thawing, samples were grown from the L1 larval stage in 25mM or 305mM NaCl conditions (grey or filled circles). At 96h±2h, five 6 cm NGM-lite plates were set up per inbred line and per NaCl treatment with 50 hand-picked embryos in either 25mM or 305mM NaCl conditions. Data points are offset in the plot for clarity. Survivorship was calculated as the number of live adults after 3 days of growth over 50, at each assay plate. Error bars indicate one standard deviation among assay plates. Data was analysed with ANOVA, where parental and offspring NaCl environment were taken as the fixed independent variables (inclusion of inbred line as a random independent variable did not change the results). ANOVA shows a significant offspring environment effect ($F_{1, 155}$=82, p-value<0.001), but no interaction between parental and offspring environments that would be indicative of maternal effects.

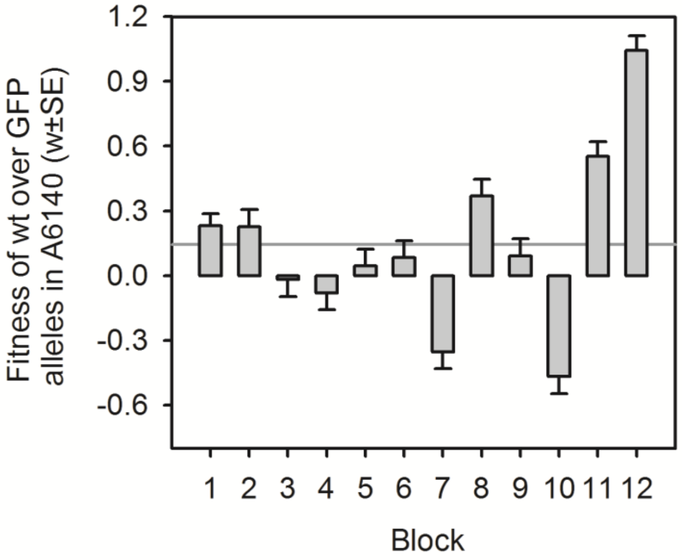

Figure S3. Fitness of wild-type over GFP alleles.

Shown are the fitness values (*w*) of A6140 alleles over A6140GFP alleles in head-to-head competitions assays in 305mM NaCl. Mean and error least-square estimates were obtained from an ANOVA, taking block as the fixed independent variable. There is great heterogeneity among blocks ($F_{11,29}$=30.5, p-value<0.001). Grey line indicates the mean fitness among blocks, as determined with a linear mixed effects model taking block as the random independent variable. There is no advantage of the wild-type over GFP alleles (total number of competitions 41, |z|=1.3, p-value=0.21).

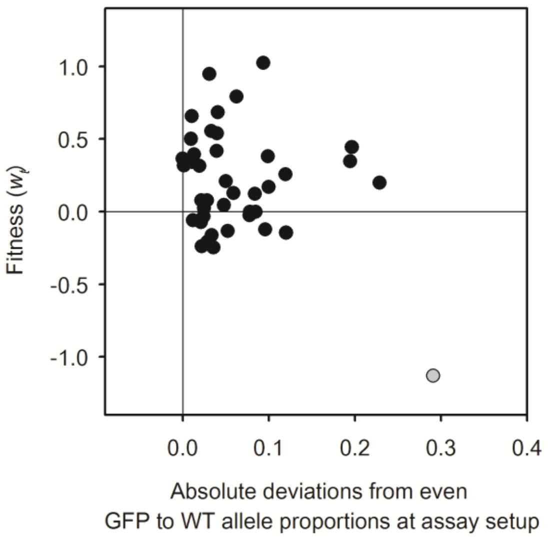

Figure S4. Quality control for fitness data.

Fitness estimates of the experimental populations obtained at a given block at 305mM NaCl were transformed by subtracting the average w of the A6140 population for the corresponding block (from Figure S3). These w_t values are plotted against the absolute deviation of GFP to wild type allele frequencies at set up of the competition assays (see Methods in main text). Since only one of these mixes was done for the 4-6 replicate competitions, there is a single w_t values per experimental population. Data from the ST2 population is an outlier (grey circle), since not only the absolute deviation from even GFP to wild type allele setup proportion is the largest but also because estimated w_t is the lowest of all populations. For these reasons, we removed ST2 fitness data from all analysis.

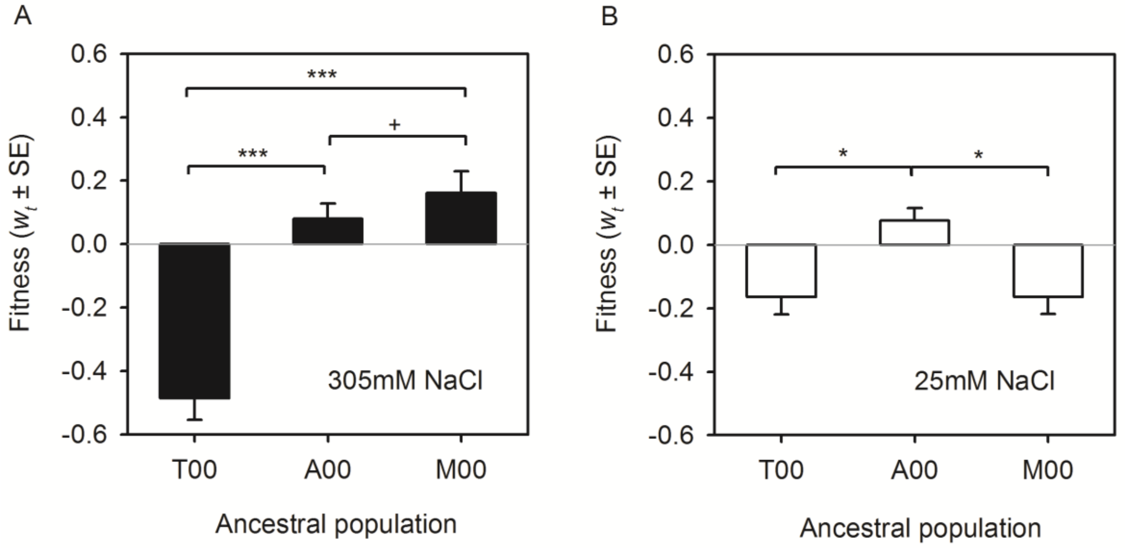

Figure S5. Fitness of the ancestral populations.

(A) Least-square fitness estimates of the three ancestral populations relative to the lab-adapted population (w_t) when the competition assays were done at 305mM NaCl. (B) w_t values at 25mM NaCl. Differences among ancestral populations were tested with ANOVA, separately for each NaCl environment. In both environments, there was significant heterogeneity among ancestral populations (305mM: $F_{2,33}$=50, p-value<0.001; 25mM: $F_{2,33}$=6.2, p-value=0.005); the significance of post hoc Tukey tests among populations are shown with +, * and *** for p-values of <0.1, <0.05 and <0.001, respectively. The trioecious population (T00) had a large fitness reduction when compared with the lab-adapted androdioecious population at 305mM NaCl (difference from zero from the ANOVA: t test=7.2, p-value<0.001) and a more modest but significant fitness reduction at 25mM NaCl (t test=3, p-value=0.004). These fitness differences reveal that the *fog-2(q71)* allele introgression is deleterious irrespective of NaCl. They also reveal the extent to which hermaphrodites can reproductive assure a dioecious population since the difference between environments is significant (separate two-tailed t test for T00 only, p-value<0.001). T00 fitness differences among environments cannot be explained by differences in sex ratios, as in both environments sex ratios are the same during the competition assays (Figure 1A, Figure S1, and results not shown). M00 was less fit than the lab-adapted androdioecious population at 25mM NaCl, revealing that the *xol-1(tm3055)* introgression was deleterious (difference from zero: t test=3, p-value=0.004). However, this was not sufficient to eliminate the advantage it has at 305mM NaCl (from the ANOVA, difference from zero: t test=2.4, p-value<0.023; separate two-tailed t test for fitness differences among environments in M00 only, p-value<0.001). This later result reveals the extent of the "cost of males" under androdioecy.

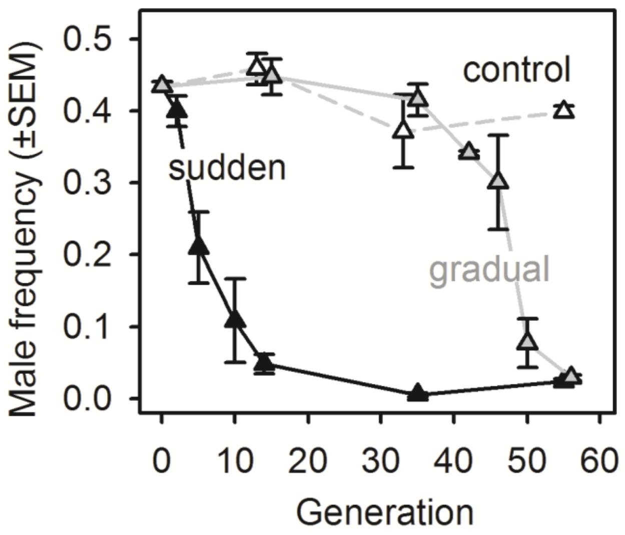

Figure S6. Evolution of male frequencies under androdioecy.

Plot shows the evolution of male frequencies in the androdioecious populations in the sudden, gradual and control regimes (Additional file 1 – Table S1). Triangles are the observed mean values of three replicate populations with error bars showing the standard mean error among them. Males were rapidly lost when populations were cultured at 305mM NaCl. In the gradual regime, male loss was complete by generation 55. Comparing these results with those of the trioecious populations, shown in main text Figure 4B, male loss was faster under androdioecy since there were no females under this reproduction system.

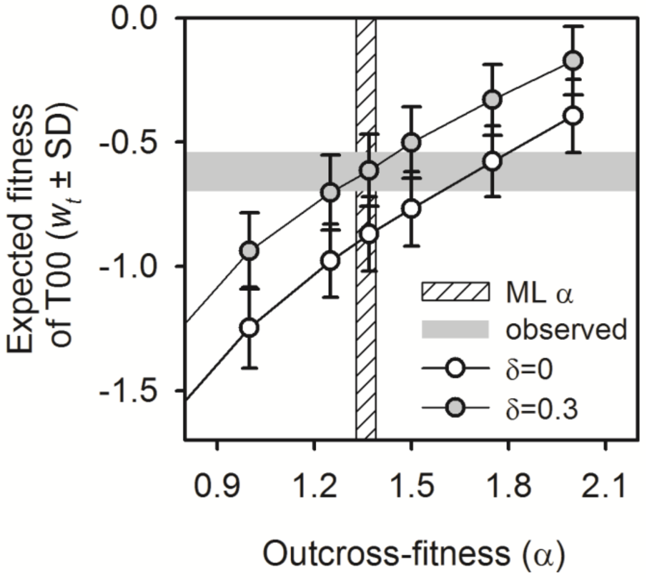

Figure S7. Expected fitness of the ancestral trioecious population.

Expected fitness values (w_t) of the ancestral trioecious population in 305mM NaCl, as a function of outcross-fitness. The confidence interval (1.96*SE) of the observed fitness is shown as a grey bar (from Additional file 1 – Figure S6), and the outcross-fitness maximum likelihood credible interval as a dashed bar (from Figure 6A). Expected w_t values were obtained by simulation of wild-type to GFP allele ratio changes over one full life-cycle head-to-head competitions between the ancestral population with the GFP tester population, following the assay protocol. Fixed even ratios were assumed at assay setup and the GFP tester population had no males. Circles indicate the mean and error bars one standard deviation among 1,000 replicate simulations. Only simulations with some inbreeding depression (δ) provided a good fit for the observed data. Illustrative δ=0.3 was included in the simulations as a sampling weight before reproduction of GFP hermaphrodites relative to wild type females, according to the *C. elegans* androdioecious model (see main text and methods). Inbreeding depression can only be due to the GFP tester population, since the ancestral trioecious population is predominantly composed of males and females and therefore little if any selfing occurs within them. This result is not incompatible with those presented in Additional file 1 – Figure S3, where the GFP allele was not deleterious relative to the wild-type allele, since both competitors were androdioecious and thus had hermaphrodites that presumably generated similar inbreeding depression. Note, however, that in main text Figure 2B there is no evidence for inbreeding depression in the ancestral androdioecious population.

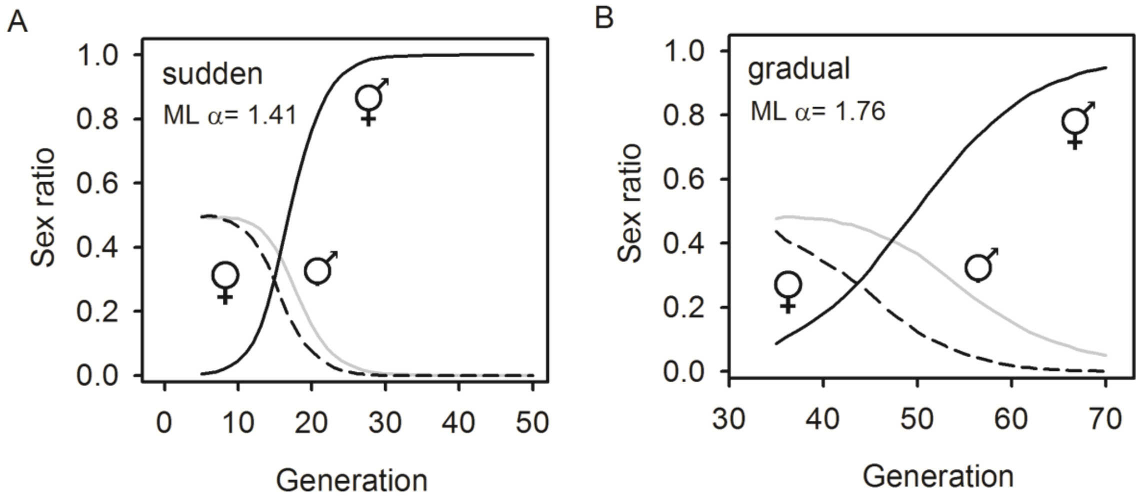

Figure S8. Expected sex ratios during transitions to selfing.

Deterministic expectations of sex ratio evolution during trioecious transitions to selfing, with the maximum likelihood outcross-fitness values (ML α) obtained for the sudden (panel A) and gradual populations (panel B). We assumed random outcrossing and selfing and no sex ratio segregation distortion (Additional file 1 – Table S2).

Table S1. ID of experimental populations and assays

			Tested for:						
ID	Reproduction system	Experimental regime	Fertility, rates	Male frequency	fog-2 genotypes	Fitness	SNP genotypes	Male fitness	Numerical modelling
A6140	Androdioecy	Lab-Adapted	x	x		x	x		
A00	Androdioecy	Ancestral		x		x			
D00	Dioecy	Ancestral	x					x	
T00	Trioecy	Ancestral		x		x			
M00	Monoecy	Ancestral				x			
CT1	Trioecy	Control		x	x	x	x		
CT2	Trioecy	Control		x	x	x	x		
CT3	Trioecy	Control		x	x	x	x		
GT1	Trioecy	Gradual		x	x	x	x		x
GT2	Trioecy	Gradual		x	x	x	x		x
GT3	Trioecy	Gradual		x	x	x	x		x
GT4	Trioecy	Gradual				x	x		
ST1	Trioecy	Sudden		x	x	x	x		x
ST2	Trioecy	Sudden				x	x		
ST3	Trioecy	Sudden		x	x	x	x		x
ST4	Trioecy	Sudden				x	x		
ST5	Trioecy	Sudden		x	x	x	x		x
ST6	Trioecy	Sudden				x	x		
ST7	Trioecy	Sudden				x	x		
CM1	Monoecy	Control				x	x		
CM2	Monoecy	Control				x	x		
CM3	Monoecy	Control				x	x		
GM1	Monoecy	Gradual				x	x		
GM2	Monoecy	Gradual				x	x		
GM3	Monoecy	Gradual				x	x		
GM5	Monoecy	Gradual				x	x		
SM1	Monoecy	Sudden				x	x		
SM2	Monoecy	Sudden				x	x		
SM3	Monoecy	Sudden				x	x		
SM4	Monoecy	Sudden				x	x		
SM5	Monoecy	Sudden				x	x		
SM6	Monoecy	Sudden				x	x		
SM7	Monoecy	Sudden				x	x		
CA1	Androdioecy	Control		x		x	x		
CA2	Androdioecy	Control		x		x	x		
CA3	Androdioecy	Control		x		x	x		
GA1	Androdioecy	Gradual		x		x	x		
GA2	Androdioecy	Gradual		x		x	x		
GA3	Androdioecy	Gradual				x	x		
GA4	Androdioecy	Gradual				x	x		
SA1	Androdioecy	Sudden		x		x	x		
SA2	Androdioecy	Sudden		x		x	x		
SA3	Androdioecy	Sudden		x		x	x		
SA4	Androdioecy	Sudden				x	x		
SD1	Dioecy	Sudden	x					x	
SD2	Dioecy	Sudden	x					x	
SD3	Dioecy	Sudden	x					x	
SD4	Dioecy	Sudden	x					x	

Table S2 – Expected reproduction table under trioecy

Breeding mode	Offspring
wt/wt, XX selfed	wt/wt, XX
wt/q71, XX selfed	1/4 wt/wt, XX + 1/2 wt/q71, XX + 1/4 q71/q71, XX
wt/wt, XX ⊗ wt/wt, XØ	1/2 wt/wt, XX + 1/2 wt/wt, XØ
wt/wt, XX ⊗ wt/q71, XØ	1/4 wt/wt, XX + 1/4 wt/q71, XX + 1/4 wt/wt, XØ + 1/4 wt/q71, XØ
wt/wt, XX ⊗ q71/q71, XØ	1/2 wt/q71, XX + 1/2 wt/q71, XØ
wt/q71, XX ⊗ wt/wt, XØ	1/4 wt/wt, XX + 1/4 wt/q71, XX + 1/4 wt/wt, XØ + 1/4 wt/q71, XØ
wt/q71, XX ⊗ wt/q71, XØ	1/8 wt/wt, XX + 1/4 wt/q71, XX + 1/8 q71/q71, XX + 1/8 wt/wt, XØ + 1/4 wt/q71, XØ + 1/8 q71/q71, XØ
wt/q71, XX ⊗ q71/q71, XØ	1/4 wt/q71, XX + 1/4 q71/q71, XX + 1/4 wt/q71, XØ + 1/4 q71/q71, XØ
q71/q71, XX ⊗ wt/wt, XØ	1/2 wt/q71, XX + 1/2 wt/q71, XØ
q71/q71, XX ⊗ wt/q71, XØ	1/4 wt/q71, XX + 1/4 q71/q71, XX + 1/4 wt/q71, XØ + 1/4 q71/q71, XØ
q71/q71, XX ⊗ q71/q71, XØ	1/2 q71/q71, XX + 1/2 q71/q71, XØ

First column shows the expected random selfing and outcrossing among males, females and hermaphrodites that occurs under trioecy. *C. elegans* is a diploid organism, whose sex determination is chromosomal with hermaphrodites and females XX, and males XØ. Hermaphrodites cannot mate with each other nor with females. The autosomal *fog-2(wt)* allele is dominant over the *fog-2(q71)* allele in the determination of hermaphroditism. The right columns shows the expected sex ratios in the offspring generation, assuming that there is no segregation distortion at either the *fog-2* locus or the X-chromosome.

Table S3. SNP sample sizes employed to calculate r^2

Population	no.SNPs
A6140	41
SA1	41
SA2	40
SA3	31
SA4	40
ST1	40
ST2	41*
ST3	45
ST4	39
ST5	38
ST6	46
ST7	41
SM1	23
SM2	30
SM3	29
SM4	1
SM5	31
SM6	14
SM7	0
GA1	37
GA2	45
GA3	46
GA4	41
GT1	38
GT2	46
GT3	44
GT5	46
GM1	41
GM2	31
GM3	37
GM5	26
CA1	46
CA2	40
CA3	45
CT1	42
CT2	48
CT3	50
CM1	30
CM2	31
CM3	35

*not included in the fitness response or SNP genotype analysis
